# Unravelling the seasonal dynamics of the metabolome of white asparagus spears using untargeted metabolomics

**DOI:** 10.1007/s11306-023-01993-0

**Published:** 2023-03-27

**Authors:** Eirini Pegiou, Jasper Engel, Roland Mumm, Robert D. Hall

**Affiliations:** 1 Laboratory of Plant Physiology, Wageningen University and Research, 6700 AA Wageningen, The Netherlands; 2 Biometris, Wageningen Plant Research, Wageningen University and Research, 6700 AA Wageningen, The Netherlands; 3 Bioscience, Wageningen Plant Research, Wageningen University and Research, 6700 AA Wageningen, The Netherlands

**Keywords:** White asparagus, *Asparagus officinalis*, Untargeted metabolomics, Seasonal dynamics, Linear modelling, Network analysis

## Abstract

**Introduction:**

The white asparagus season lasts 4 months while the harvest period per field is 8 weeks. Different varieties are better suited for harvesting early or late in the season. Little is known of the dynamics of secondary metabolites of white asparagus during the production season.

**Objective:**

Characterization of the metabolome of white asparagus spears covering volatile and non-volatile composition in relation to quality aspects.

**Methods:**

Eight varieties, harvested repeatedly during two consecutive seasons were analysed following an untargeted metabolomics workflow using SPME GC–MS and LC–MS. Linear regression, cluster and network analyses were used to explore the profile dynamics, unravel patterns and study the influence of genotype and environment.

**Results:**

The metabolite profiles were influenced by the harvest moment and genetic background. Metabolites that significantly changed over time were distributed across seven clusters based on their temporal patterns. Two clusters including monoterpenes, benzenoids and saponins showed the most prominent seasonal changes. The changes depicted by the other five clusters were mainly ≤ 2-fold relative to the harvest start. Known asparagus aroma compounds were found to be relatively stable across the season/varieties. Heat-enhanced cultivation appeared to yield spears early in season with a similar metabolome to those harvested later.

**Conclusion:**

The dynamics of the white asparagus metabolome is influenced by a complex relationship between the onset of spear development, the moment of harvest and the genetic background. The typical perceived asparagus flavour profile is unlikely to be significantly affected by these dynamics.

**Supplementary Information:**

The online version contains supplementary material available at 10.1007/s11306-023-01993-0.

## Introduction

Asparagus (*Asparagus officinalis*) is a perennial crop, the shoots (spears) of which are mainly harvested during the spring and early summer months. Different varieties have been bred to be better suited for harvest at the beginning or end of the crop season and, for logistical reasons, there are often only one or two varieties cultivated in each field. The usual harvest period for each field is 8 weeks, during which spears are harvested every other day. Thereafter, the plants are allowed to produce leafy shoots as normal in order to support root crown development and nutrient reserve accumulation for the following year. Based on these different parameters, growers plan their harvest regime to be able to provide products continuously over a ca. 4-month period (Pegiou et al., 2020).

The metabolite composition of plants is highly linked to the developmental stage, growth rate and genotype as well as to response mechanisms to abiotic and biotic stress. Changes in the metabolite profiles of crops underpin these processes and may also have significant impact on crop quality (e.g., texture, colour, flavour) (Hall, 2006; Jaramillo et al., 2007; van Treuren et al., 2018). During the asparagus season—for example in North Europe between March and June (Pegiou et al., 2020)—temperature fluctuations and weather changes regularly occur. Asparagus growers have developed cultivation method modifications not just to protect their crops from the uncontrollably changing weather conditions which could be detrimental to the spear yield and quality (Heißner et al., 2006), but also to enable earlier spear development than usual. Although not severe, the typically varying climatological conditions during the asparagus season might lead to metabolic changes of relevance to specific crop quality traits. Plastic mini-tunnels are regularly used but more impactful modifications such as using a warm water soil heating system (heated fields) or the use of greenhouses can be applied (Pegiou et al., 2020).

The quality of asparagus spears is strongly connected to the multitude of secondary metabolites formed during development (Pegiou et al., 2020). One important aspect of asparagus quality is its flavour and the metabolites that significantly contribute to the asparagus flavour profile comprise chemically-diverse molecules. These include the sulphur-containing compounds dimethyl sulphide, methional and asparagusic acid as well as 2-methoxy-3-isopropyl pyrazine, medium-chained carbonyls such as hexanal and 1-octen-3-ol, and bitter-related compounds such as saponins and flavonoids (Dawid & Hofmann, 2012; Hoberg et al., 2008; Kawano et al., 1975; Ulrich et al., 2001).

Changes during the asparagus metabolome throughout the harvest period have so far been poorly studied. Using green asparagus, Soteriou et al. (2021) showed a decrease in total sugars and an increase in total phenolics during the harvest season. Earlier, using white spears, Creydt et al. (2018) studied the profile of secondary non-volatile compounds and the variation in materials harvested in various European countries and from different years. They suggested that there is a strong influence of both the local climate and specifically the temperature, and soil composition on asparagus metabolites. In a comparative metabolomics study using three asparagus varieties in both green and white forms, grown at a single field location and harvested at two time-points within one season, Pegiou et al. (2021) showed that even a 3-week harvest interval can have significant influence on the composition of secondary metabolites although the genetic background effect was minor. Altogether, these initial studies indicate that the metabolome of white asparagus is indeed influenced by environmental factors (e.g., harvest time-point, meteorological conditions, geographical location). However, limited knowledge is available regarding the genetic background effect, temporal changes of the secondary metabolome of asparagus spears and their interactions which may occur during the complete harvest period and which are of potential sensory relevance.

The central aim of the study presented here was to explore thoroughly the metabolite composition of white spears during the harvest season. It was hypothesized that the majority of secondary metabolites would show temporal changes and we aimed to investigate the extent of these temporal patterns. Complementary metabolomics platforms were used to analyse white spears of eight varieties grown and harvested at different times across the total asparagus season (early, middle and late). Spears were harvested in two consecutive seasons (2019 and 2020), from selected fields in the Netherlands where also contrasting cultivation methods were applied. We focused on the analysis of volatile and semi-polar non-volatile metabolites as both groups comprise compounds which are highly important for flavour and quality (e.g., Pegiou et al. 2020, 2021). In particular we explored the impact of environmental (harvest time-point, cultivation method) and genetic (variety) factors on the dynamics of the biochemical composition of the spears using the extensive 2019 data. Material from 2020 was analysed to validate observations made in 2019.

## Materials and methods

### Asparagus material

White spears of eight varieties were kindly provided by the commercial grower Teboza BV (Limburg, The Netherlands). All varieties were grown following standard commercial cultivation practices, as described below. All fields had been enriched with natural fertilizers to ensure optimal nutrient concentrations and pH adjusted for the healthy growth of the crop. Spears were harvested from fields located in the South of the Netherlands in Limburg and North Brabant, all within a 25 km radius. For every sample, the variety, exact cultivation and harvest history could be unambiguously assigned. Post-harvest treatment of all spears was standardized. The varieties were chosen based on their growing phenology and included so-called early varieties (i.e. Avalim, Gijnlim, Fortems, Cumulus), harvested mainly in the beginning to middle of the season and late varieties (i.e. Backlim, Grolim, Primems, Tallems) harvested middle-end of the season. The four cultivation methods applied were (1) the standard open field method, (2) using mini-tunnels, (3) heated fields and (4) a greenhouse. The last two methods were applied specifically when the aim was to harvest spears artificially early in the season. In all cases, asparagus beds were covered with an opaque plastic foil to avoid exposure of the spears to sunlight following standard production practice.

Each variety was sampled at 3–8 time points from one specific field and the harvesting scheme, in both years, was arranged based on the planning for each variety, field and the grower’s advice (Supplementary Information; Table S1). On each sampling day, asparagus spears were freshly harvested and within 2–3 h, transported on ice to the lab. Then the spears were carefully rinsed with cool water and further treated as described previously (Pegiou et al., 2021). Three biological replicates were generated, each consisting of three spears per variety and per harvest day (2019: 123 biological samples; 2020: 57 biological samples). Subsequently, the spears were cut into pieces, ground in liquid nitrogen, and powders were stored at − 80 °C until further analysis (Pegiou et al., 2021).

### Untargeted metabolomics

#### Analysis of volatile compounds

Solid-phase microextraction gas chromatography mass spectrometry (SPME GC–MS) was applied to collect the volatile metabolite data as described previously (Pegiou et al., 2021). For this, 0.5 g frozen asparagus powder was mixed with 0.73 g CaCl_2_ dihydrate (Sigma-Aldrich, The Netherlands) and 0.5 mL 0.09 M EDTA-NaOH (Sigma-Aldrich, The Netherlands ) (pH 7.5) in a pre-cooled 10  mL ND18 headspace glass vial (BGB®, Germany). Vials were closed with magnetic screw caps (8 mm hole) with Silicone/PTFE septa (BGB®, Germany). A mix of all sample powders was prepared and 0.5 g aliquots were used as quality control (QC) samples which were analysed together with the biological samples. A series of *n*-alkanes (C_6_–C_21_) (Sigma-Aldrich, the Netherlands), was analysed with the same SPME GC-MS method to calculate retention indices (RIs).

#### Analysis of non-volatile compounds

Ultra-performance liquid chromatography MS (LC–MS) was performed for the profiling of the semi-polar non-volatile compounds, The semi-polar compounds from each sample were extracted by mixing 0.3 g frozen powder with 0.9 mL 32.04 M methanol containing 0.035 M formic acid (Sigma-Aldrich, The Netherlands) followed by sonication and centrifugation, as described previously by De Vos et al. (2007). The LC–MS calibration and analysis settings were as previously (Pegiou et al., 2021). A mix of all biological samples was prepared and 0.3 g aliquots were used as QC samples and analysed together with the biological samples.

#### Metabolomics data processing

All samples were analysed using both analytical platforms. GC–MS analyses were performed in 4–6 batches of 32 injections each. For LC–MS, samples were analysed in a single sequence of 2 batches. Per sequence or batch, 4–6 QCs were analysed. Raw data were processed following the established untargeted metabolomics workflow using the software packages MetAlign and MSClust (De Vos et al., 2007; Lommen, 2009; Tikunov et al., 2012). After baseline correction, peak picking and alignment of the mass signals, mass features that were present in more than two biological replicates for each sample were retained. Subsequently, mass signals were reconstructed into potential compounds based on their correlation across retention time and the intensity pattern across samples. The relative abundances of the reconstructed compounds in the processed GC–MS and LC–MS data were log2-transformed and a correction for between-batch differences and within-batch signal drift was carried out, based on the QCs (Wehrens et al., 2016).

#### Metabolite identification

Volatile metabolites were identified by matching the obtained reconstructed mass spectra and calculated RIs with those of authentic reference standards and using the NIST17 Mass Spectral library and in-house databases. Non-volatile compounds were putatively identified by matching their molecular ion mass and other in-source fragments with those in online databases KnaPSAck (http://www.knapsackfamily.com/), and mzCloud (https://www.mzcloud.org/) and in previous reports on asparagus materials (Dawid & Hofmann, 2012, 2014; Nakabayashi et al., 2015; Pegiou et al., 2020, 2021). Levels of identification (LOI) were assigned following the Metabolomics Standards Initiative guidelines (Sumner et al., 2007). LOI 1 indicates metabolites that were confirmed with authentic reference standards and LOI 4 indicates unidentified compounds (unknowns). For volatile compounds, LOI 2 indicates compounds with average EI-MS spectral match > 800 and RI error tolerance < 15, while LOI 3 indicates compounds for which one of the mentioned conditions applies. For non-volatile compounds, LOI 2 indicates compounds of which the calculated monoisotopic mass matched the reference with error < 5 ppm, while LOI 3 indicates compounds of which the calculated monoisotopic mass matched the refence with error > 5 ppm.

### Statistical analysis and visualization tools

Statistical analysis and combining analyses outputs for visualization of data was performed using RStudio with R version 4.0.3 (2022.02.3+492), Microsoft Excel and PowerPoint for Microsoft 365 (version 2104, 2021).

The metabolite profiles were investigated by hierarchical clustering (HCA) of the compounds using a correlation-based distance and average linkage. The clustering of the temporal patterns was visualized in a heatmap displaying for each variety, harvest week, and cultivation type the average abundance of a compound, relative to the 1st sampled harvest week for that variety, i.e. foldchange (FC). FC was not computed for 7 volatiles and 50 non-volatiles due to ≥ 90% missing values. In the heatmap, compounds (rows) were ordered according to the HCA outcome, while varieties and harvest weeks within a variety (columns) were placed in chronological order. Additional to HCA, the variation between the metabolite profiles was explored by principal component analysis (PCA) of the Pareto-scaled data matrix. HCA and PCA were performed using R packages *dendextend*, gplots, *ggplot2* and basic R functions.

To investigate the temporal patterns for each compound in more detail, a subset of the data (varieties harvested at least 6 times in 2019) was studied by applying linear regression models. The linear model comprised fixed effects for genetic background (variety), harvest moment (time) and the interaction between variety and time. Time-trends were fitted for each variety using a natural spline with 3 degrees of freedom. For each variety, the temporal profiles were expressed in the model as logFC from the starting harvest time-point onwards and were labelled based on sampling week i.e. week 1–8. The significance of the effects (variety, time, interaction) was assessed by moderated *F*-tests, taking into account the relationship between the mean and the variance of the metabolite abundances (Ritchie et al., 2015). A False Discovery Rate (FDR) correction was applied to the *p*-values. Adjusted *p*-values < 0.05 were considered significant. Linear regression analysis was performed using the R package *limma* (Ritchie et al., 2015).

The estimated temporal profiles of a set of metabolites were grouped by cluster analysis. The set comprised all compounds for which a significant effect of time had been detected by the linear model. Groups of highly correlated metabolites were identified by weighted correlation network analysis (WCNA) using a topological overlap (TOM)-based measure of the dissimilarity between the estimated temporal profiles (that were concatenated across varieties). TOM incorporates information on the direct association (correlation) between pairs of metabolites and information on their neighbourhood (interconnectedness) in a metabolite network that is estimated from the data. Consequently, it is considered a robust approach to assess the association between compounds and counteract the effects of spurious correlations (Yip & Horvath, 2007). Clustering by WCNA was performed in R package WGCNA (Langfelder & Horvath, 2008) selecting the “signed” network option (only positively associated compounds clustering together), with the minimum module (cluster) size set to 10, and deepSplit = 2, minKMEtoStay = 0.5, and mergeCutThrehold = 0.25. Since scale-free-topology could not be achieved, the option softPower was set to a default of 12. Note that similar results were obtained by grouping metabolites using average-linkage HCA with a correlation-based dissimilarity measure (not shown).

To investigate potential influence on the asparagus flavour, the fitted time-trends of compounds that have been previously proposed to be sensory-relevant for white asparagus materials were examined in more detail, independent of their significance in the *F*-test. These were dimethyl sulphide, dimethyl disulphide, methanethiol, methional, hexanal, 2-hexenal, 1-octen-3-ol, 2,3-pctanedione, 2-methoxy-3-isopropyl pyrazine, 2-methoxy-3-isobutyl pyrazine, 2-pentylfuran (Ulrich et al., 2001), asparagusic acid (Dawid & Hofmann, 2012; Tressl et al., 1977b) and its derivative asparaptine (Nakabayashi et al., 2015), protodioscin, shatavarin IX (Dawid & Hofmann, 2014), rutin (Hoberg et al., 1999), and 3-feruloylquinic acid which is derivative of ferulic acid (Rodrĩguez-Arcos et al., 2004; Tressl et al., 1977a).

The influence of the genetic background and cultivation method on the metabolome was studied by a linear model with a fixed effect for each variety and cultivation combination. The model was applied to subsets of the data. Each subset consisted of all varieties harvested at one specific time-point. Significance testing by moderated *F*-tests was carried out analogously to the procedure described above.

## Results

### Composition of the white asparagus metabolome

Following an untargeted metabolomics workflow used to process the acquired GC–MS and LC–MS data, 105 volatile and 261 non-volatile compounds were detected. Among the 89 GC–MS and 58 LC–MS metabolites which could be annotated were compounds from various classes including aldehydes (10% of the annotated compounds), alcohols (7%), flavonoid glycosides (18%), furans (5%), monoterpenes (10%), pyrazines (1%), saponins (10%), sulphur-containing compounds (16%) and some amino acids and sugars (12%) (Supplementary Information; Tables S2, S3). PCA of the metabolite profiles of all varieties demonstrates that the variation between the QCs was less than the overall variation between the biological samples, indicating good technical reproducibility (Supplementary Information; Fig. S1a, b).

**Fig. 1:**
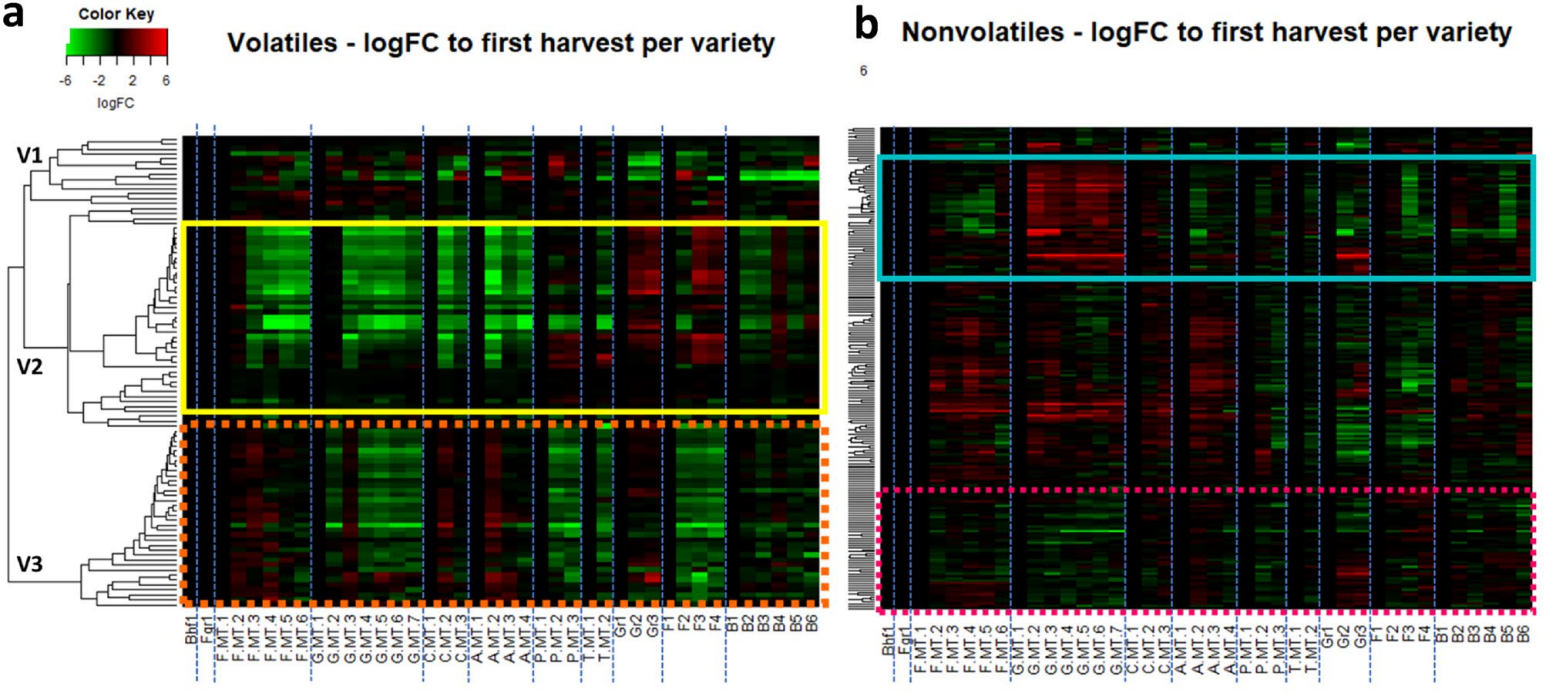
Heatmap and HCA of the time-trends of the abundances (rows) of volatile (**a**) and non-volatile (**b**) metabolites of white asparagus spears harvested in 2019. The abundance of each metabolite at each harvest week is expressed as a log2-foldchange relative to the first harvest time-point per variety. LogFC values have been truncated to a specific range (− 6 to 6) for consistency. Columns represent consecutive harvest weeks per variety and the different varieties are separated by dotted blue lines. Varieties are ordered in chronological order of their overall seasonal harvest period. *Bhf* Backlim heated field, *Fgh* Fortems greenhouse, *F.MT* Fortems mini-tunnel, *G.MT* Gijnlim mini-tunnel, *C.MT* Cumulus mini-tunnel, *A.MT* Avalim mini-tunnel, *P.MT* Primems mini-tunnel, *T.MT* Tallems mini-tunnel, *Gr* Grolim, *F* Fortems open field, *B* Backlim open field. Numbers indicate the harvest time-point per variety. Green indicates a decrease and red indicates an increase in metabolite abundance compared to the first harvest. Dendrograms show the clustering of the metabolites by HCA using correlation-based distance and average linkage. **a**Yellow solid line box highlights the majority of clustered compounds in V2. Orange dotted-line box highlights clustered compounds in V3. **b** Turquoise box highlights clustered compounds in N2. Pink dotted-line box highlights clustered compounds in N4

To explore the abundance of the detected compounds across the harvest season, the profiles of the volatile and non-volatile metabolites were examined by HCA. To assist in the investigation of potential temporal patterns, the log2-transformed foldchanges (logFC) of the abundances of each metabolite were visualized in a heatmap where each column is 1 harvest week of one variety and varieties are in chronological order of their harvest period (Fig. 1). The abundance of most metabolites (rows) appeared to change during the 2019 season as indicated by a colour change between the different harvest moments per variety (columns). The time-trends of the majority of metabolites appeared to differ between varieties throughout the harvest period. The volatile metabolites were distributed across three main clusters based on the formed dendrograms of the cluster analysis (Fig. 1a). Compounds in cluster V1 (ca. 10% of the total) changed over time without particularly showing a general or variety-specific trend. The majority of the volatiles in cluster V2 (ca. 40% of the total), decreased across time for all the early varieties Fortems, Gijnlim, Cumulus and Avalim which were grown in a ‘mini-tunnel’, as demonstrated by the intense green colour (Fig. 1a; yellow box). In contrast, for the late varieties Grolim, Fortems and Backlim grown in a regular ‘open field’, these same V2 volatile compounds generally followed an increasing trend towards the end season (but with a fall-off for Backlim). The abundances of the volatile compounds in cluster V3 (ca. 40% of the total) showed an increasing time-trend for Fortems, Cumulus, and Avalim grown in ‘mini-tunnels’, as indicated by the later intense red colour, while these generally decreased along the harvest period of Gijnlim, Primems and the ‘open field’-cultivated Fortems (Fig. 1a; orange dotted-line box).

Heterogeneity in the temporal changes was also observed with respect to the non-volatile metabolites which seemed to be distributed across four main clusters based on the dendrograms (Fig. 1b). The trends of the majority of compounds in clusters N1 and N2 (ca. 20% of the total) were similar and increased across time specifically for Gijnlim (Fig. 1b; turquoise box). The abundances of most compounds in N3 (ca. 45% of the total) showed a mid-season peak for the early ‘mini-tunnel’ varieties Fortems, Gijnlim, Cumulus and Avalim while these metabolites tend to show a gradual decrease during the harvest period for late varieties Primems grown in mini-tunnel and Grolim and Fortems grown in open field (Fig. 1b). The trends of most compounds in N4 (ca. 20% of the total) were decreasing for Gijnlim in particular, but varied a lot for the other varieties (Fig. 1b; pink dotted-line box).

The various trends observed for both volatile (Fig. 1a) and non-volatile compounds (Fig. 1b) indicated a complexity with respect to the seasonal dynamics of the secondary metabolome of white asparagus spears. The composition of the volatile profiles appeared to be influenced to some extent by the time of harvest (Supplementary Information; Fig. S1c; yellow arrow). Regarding the non-volatile profiles, it appeared that Backlim, grown both in the open field and heated field, had a different composition to the other varieties (Supplementary Information; Fig. S1d, e; red circles). To unravel further and assess the significance of the observed complex dynamics, more advanced data mining approaches were followed.

### The seasonal dynamics of the asparagus metabolome

The explorative HCA and PCA of the metabolite profiles indicated that the harvest moment during the crop season and the genetic background are of potential relevance regarding the dynamics of the metabolome. The temporal profiles of all detected compounds were modelled for the standard 8-week harvest period. We focused on varieties sampled at least 6 times including the first and final week of their harvest period. These were therefore, Backlim (open field), Fortems (mini-tunnel) and Gijnlim (mini-tunnel) (Table S1a). The rest of the 2019 and the full 2020 data sets were subsequently examined for the potential validation of our findings.

**Fig. 2:**
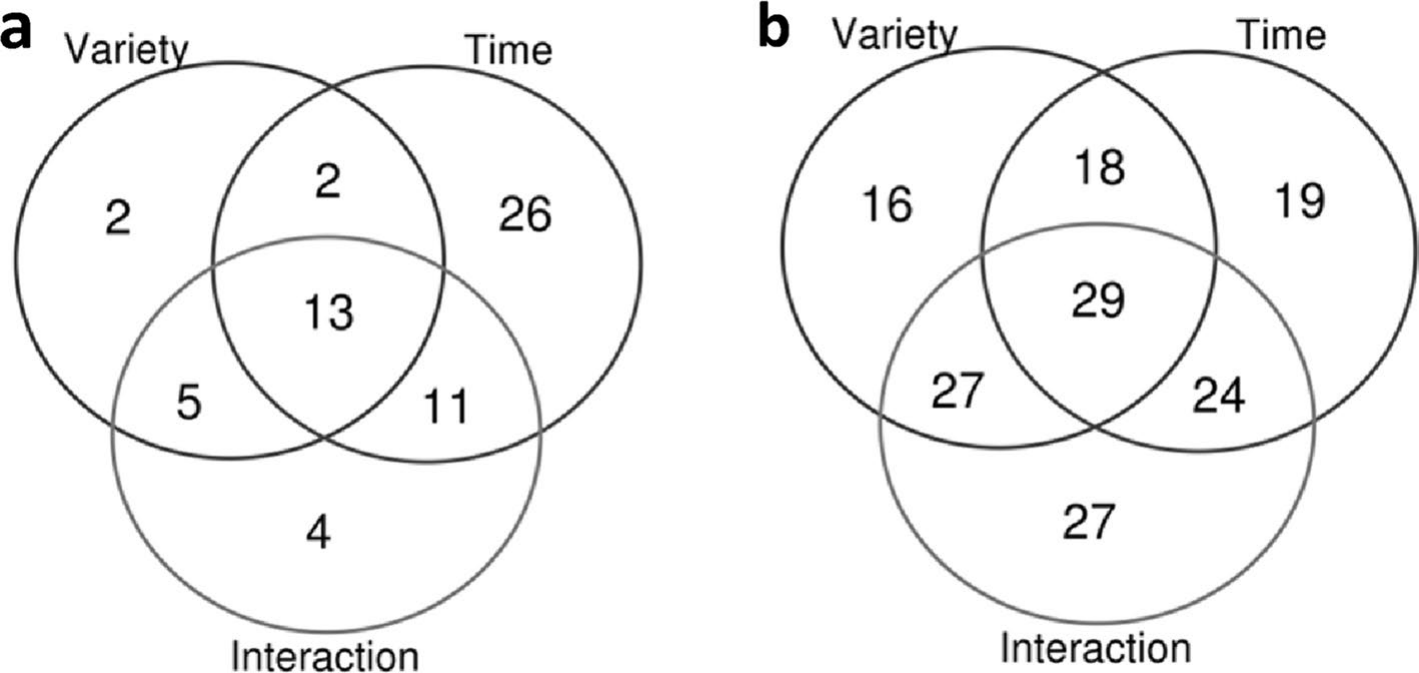
Venn diagrams of the number of **a** volatile and **b** non-volatile compounds that were significantly different between the three varieties at the start of their harvest period (‘Variety’), the abundance of which was at least at one time-point significantly different compared to the first harvest moment (‘Time’), and the time-trends which were significantly varying between the three varieties (‘Interaction’). The lists of specific compounds can be found in Tables S4a (volatiles) and S4b (non-volatiles)

Compounds which significantly changed over time regardless of the genetic background (significant time effect, but non-significant interaction) were examined, as were compounds which changed following variety-specific trends (Interaction effect). The metabolites detected at significantly different levels between the three varieties at the start of their harvest are highlighted by the ‘Variety’ effect (Fig. 2) and these are discussed further in later parts of this article.

The first analysis highlighted 28 volatiles (25% of all volatiles) and 37 non-volatiles (14% of all non-volatiles) which significantly changed over time throughout the harvest period in all three varieties (Fig. 2; Time but not Interaction). Among these compounds were carbonyls with 6–8 carbon atoms (e.g., hexanal, (*E*)-2-hexenal, heptanal, 1-octen-3-one), sulphur-containing compounds and two saponins (Supplementary Information; Table S4). The time-trends of 33 volatiles and 107 non-volatiles were found to be variety-specific (Fig. 2; Interaction) and these compounds were mainly monoterpenes, benzenoids and saponins (Table S4). The profiles of approximately 40% of the volatile (42) and non-volatile metabolites (103) were not significantly affected by either the genetic background or harvest time-points. Among these were unsaturated medium-chained aldehydes (e.g., (*E*,*E*)-2,4-heptadienal and (*E*)-2-decenal), two sulphur-containing compounds (i.e. dimethyl disulphide and *S*-adenosylhomocysteine), 2-pentylfuran, one kaempferol glucoside (772.20662 Da), a few sugars and amino acids.

**Fig. 3:**
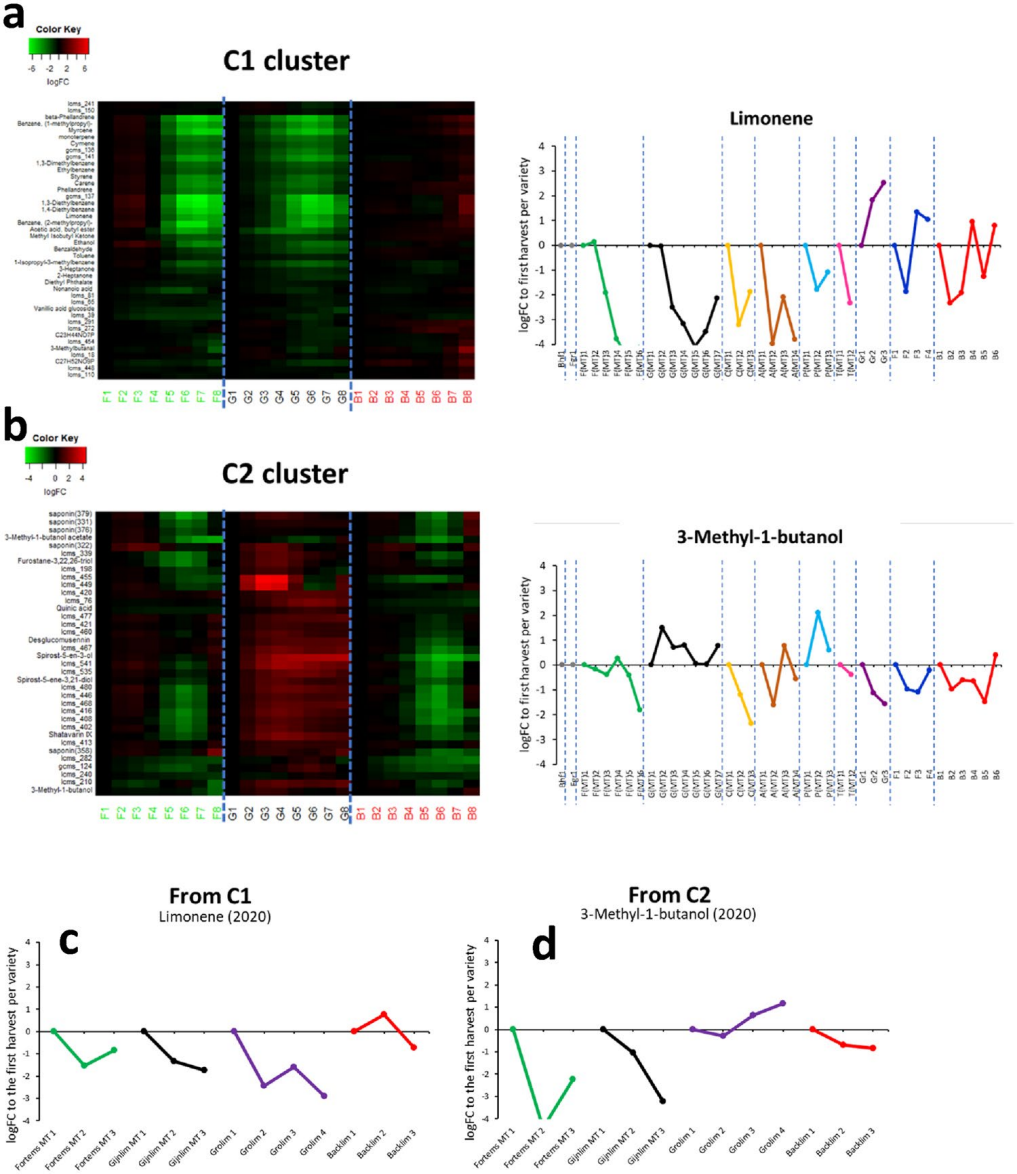
Weighted correlation network analysis of the modelled time-trends of metabolites detected in white asparagus spears harvested in 2019. Metabolites were clustered based on their fitted time-trends using topological overlap as a distance measure. Two clusters (C1 and C2) of the seven that were formed and the time-trends of the clustered metabolites (rows) have been visualised in a heatmap. Each column represents 1 harvest week of one variety. Varieties are ordered in chronological order of their overall seasonal harvest period. Vertical blue dotted-lines separate the harvest periods of the varieties (F: Fortems, G: Gijnlim, B: Backlim and numbers indicate the harvest time-point). Clusters C1 (**a**) and C2 (**b**) comprised metabolites for which the time-trends showed the largest changes over time. A representative metabolite per cluster was examined with respect to the non-modelled time-trend for all eight varieties labelled same as in Fig. 1 (*line graphs in a and b*). LogFC values in y-axis of line graphs have been truncated to a specific range (− 4 to 4) for consistency. The other five clusters are presented in Fig. S2 and all clusters are summarized in Table 1. Material from the 2020 crop season was analysed for validation of the findings (**c**, **d**). Lines in **c** and **d** are coloured according to variety name as in **a** and **b** (green: Fortems, black: Gijnlim, purple: Grolim, red: Backlim). Numbers indicate the sampled harvest time-point

To investigate the prominent temporal patterns in detail, the focus was given to those 61 volatiles and 144 non-volatiles that significantly changed throughout the season for at least one variety (Time and/or Interaction effects in Fig. 2a, b). The modelled time-trends (expressed as logFC relative to the first harvest) of these compounds were clustered based on TOM by applying WCNA. As, time-trends of the varieties were concatenated before WCNA, metabolites were clustered based on the similarity of their time-trends per variety, i.e. metabolites “behaved” the same over time within varieties, but the temporal patterns between varieties were not necessarily the same. This resulted in seven clusters (C1–C7) across which 169 of the 205 metabolites were distributed. The other 36 metabolites did not end up in any of the clusters, i.e. the time patterns in these metabolites were non-significant according to TOM. Most metabolites ended up in clusters C1 (42 compounds) and C2 (36 compounds) and the time-trends depicted in these clusters changed the most during the season compared to the other clusters (Fig. 3a, b). Metabolites in C1 including monoterpenes, benzenoids and some lipid products significantly decreased (≤ − 4 logFC) towards the end season for the relatively early varieties Fortems and Gijnlim grown in ‘mini-tunnels’, while they showed a slight increase at the season end for the late variety Backlim grown in open field (Fig. 3a). Metabolites in C2 comprised mainly saponins but also two volatile compounds (3-methyl-1-butanol and its acetate) and their trends were increasing specifically for Gijnlim (Fig. 3b). The compounds in the other five clusters (C3–C7) showed temporal patterns which were sometimes variety-specific, or showed only small changes (± 2 logFC) across the season (Supplementary Information; Fig. S2a–e). However, the abundances of some of those compounds followed a noticeable variety-specific trend. For example 1-methoxy-2-propanol and its acetate which clustered in C4 both notably decreased (< − 4 logFC) across the season for Backlim (Supplementary Information; Fig. S2b). The metabolites clustered in C5 included medium-chained volatile carbonyls and although they did not change more than 2-fold compared to the harvest start, their intensities decreased across the season for Gijnlim and Backlim (Supplementary Information; Fig. S2c). To help with the interpretation of these clusters, representative metabolites from each cluster and the actual (non-modelled) trends of their intensities in all of the eight studied varieties were examined (line graphs in Fig. 3a, b and Supplementary Information; Fig. S2a–e). These analyses indicated a distinction with respect to temporal patterns of metabolite composition between the early and late varieties and these are summarized in Table 1.

**Table 1:**
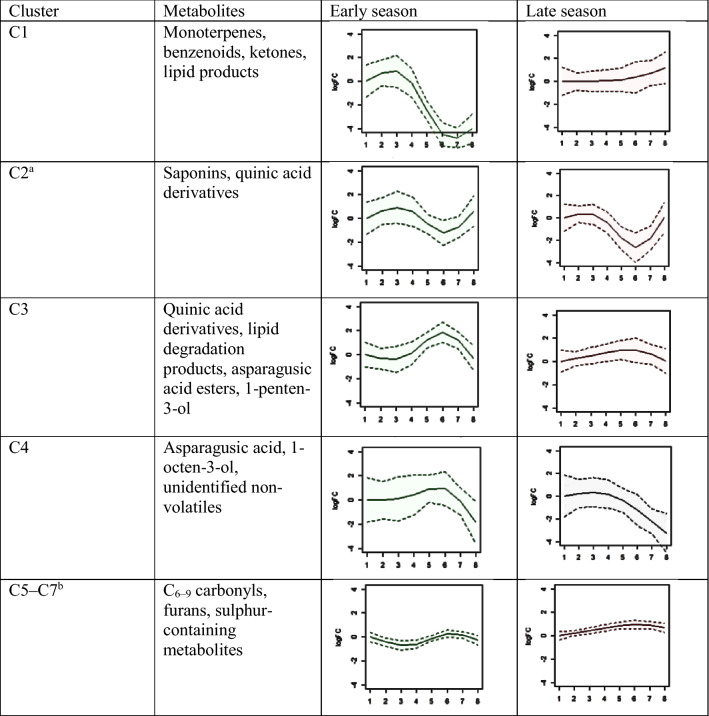
Overview of the dynamic character of asparagus metabolites during the 2019 crop season organised in 7 clusters determined by WCNA

The observed temporal patternsof the clustered metabolites are demonstrated by a representative modelled trend for the standard 8-week harvest period. Early season (variety) corresponds to a harvest period starting in March/April and late season corresponds to a harvest period starting in April/May. Trends (solid lines) are expressed as log2 foldchanges (logFC) relative to the first harvest moment. Dotted lines show 95% confidence intervals of the modelled trends

^a^Gijnlim showed a distinct pattern (increasing and then levelling off)

^b^Changes < 2fold compared to start of the harvest period

Material from the second harvest season was analysed using GC–MS and the PCA reflected and confirmed the influence of the harvest moment on the volatile profiles of asparagus spears as observed in the previous year’s samples (Supplementary Information; Fig. S3a). Most of the observed time-trends could be validated and especially the prominent ones regarding the compounds clustered in C1 (Fig. 3c) but also the subtle changes (± 2 logFC) for the volatiles clustered in C3–C7 (Supplementary Information; Fig. S4). However, the Gijnlim-specific increasing trend for compounds that were clustered in C2 was not observed in the second season (Fig. 3d).

### The potential impact of seasonal dynamics on the flavour profile

**Fig. 4:**
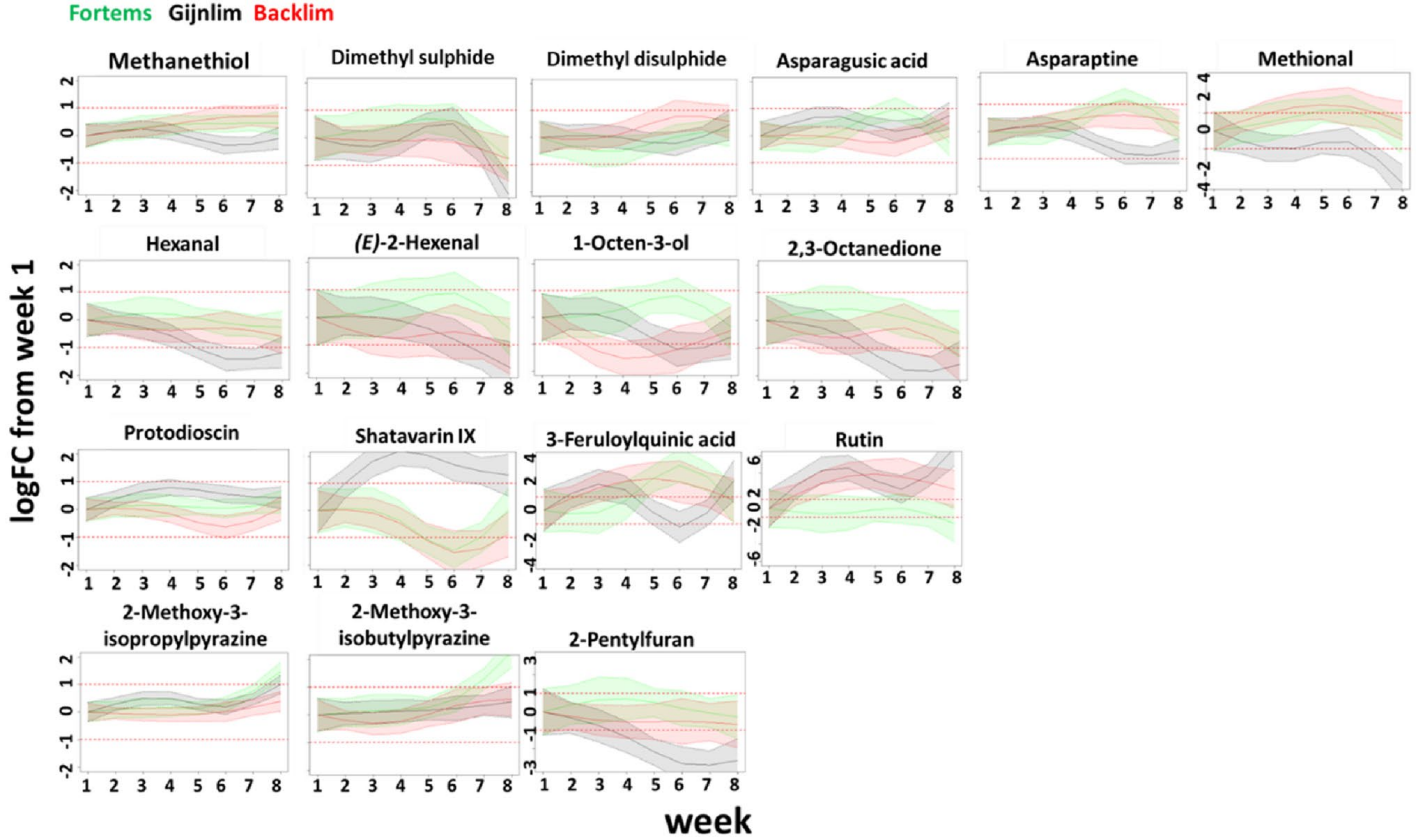
Modelled time-trends of selected flavour-relevant asparagus metabolites for the standard 8-week harvest period. The abundance of each metabolite at each harvest week is expressed as log2-transformed fold change relative to the first time-point of the harvest period per variety (green: Fortems; black: Gijnlim; red: Backlim). The 95% confidence intervals of the modelled time-trends are depicted (tinted coloured areas above and below time-trends). The horizontal red dotted lines indicate a 2-fold change relative to the first harvest moment

To obtain some insights into whether the observed overall seasonal changes may have some impact on the flavour of the spears, we also specifically looked into a number of compounds already proposed in the literature to influence the characteristic asparagus flavour. In this way, we aimed to be able to predict the potential impact of the observed changes in metabolite composition on the asparagus flavour dynamics throughout the harvest period. The modelled 8-week trends from Backlim, Fortems and Gijnlim harvested in 2019 were used to follow the temporal patterns of 17 sensory-relevant metabolites (Fig. 4). The regression analysis showed that dimethyl sulphide, methional, asparagusic acid, asparaptine, hexanal, (*E*)-2-hexenal, 1-octen-3-ol, 2,3-octanedione, shatavarin IX and 3-feruloylquinic acid significantly change over time for at least one of the varieties (Table S4). The time-trends of these compounds, except for shatavarin IX (C2) and 3-feruloylquinic acid (C3) clustered in either C4, C5 or C7 clusters (Supplementary Information; Fig. S2) indicating shifts which were limited within a 2-fold change compared to the start of the harvest period. The changes of most of these potentially sensory-relevant metabolites were indeed ± 2FC (Fig. 4). Moreover, no significant differences between varieties were found specifically for dimethyl sulphide, dimethyl disulphide, asparagusic acid, hexanal, (*E*)-2-hexenal, 2,3-octanedione, 2-methoxy-3-isopropyl pyrazine and 2-methoxy-3-isobutyl pyrazine (Fig. 4, Table S4a).

### The influence of genetic background on asparagus metabolome

**Fig. 5:**
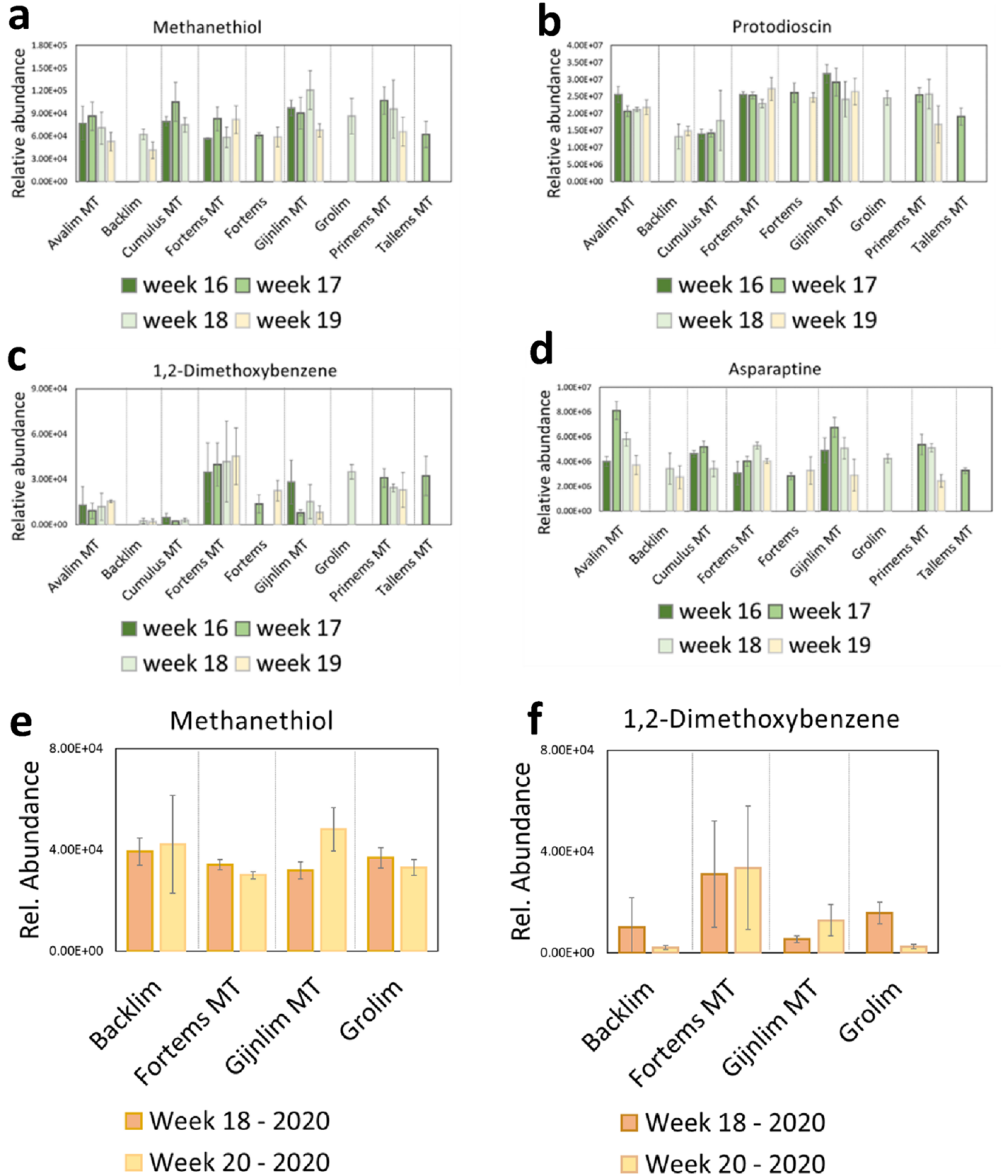
Abundance of selected metabolites in different white asparagus varieties harvested in different calendar weeks in 2019 (**a–d**) and 2020 (**e**, **f**). The selected metabolites were found to be significantly different between varieties but either, without changing over time within each variety (methanethiol, protodioscin) or also changing over time (1,2-dimethoxybenzene, asparaptine). Missing bars in **a**–**d** indicate that the variety was not harvested in that week

The genetic background of white asparagus spears also appeared to influence the metabolome. We examined the compounds that significantly varied between varieties either at the beginning or throughout the harvest period (Variety and/or Interaction effects in Fig. 2a, b). These comprised 7 volatiles (methanethiol, 3-methylbutanal, 1,2-dimethoxybenzene and four unidentified) and 43 non-volatiles (including saponins, asparaptine, quinic acid) as well as 16 other compounds which did not significantly change over time (Table S4). We compared the abundances of these compounds between varieties harvested on the same day, and this was repeated for several time-points (calendar weeks 16–19). Varietal differences were observed as demonstrated for example by methanethiol (Fig. 5a) and protodioscin (Fig. 5b) when comparing the bar graphs of the same colour, while no significant differences were indeed found between the harvest moments of each variety. The variety-specific compounds which also changed during the season were examined and the patterns were verified as exemplified by 1,2-dimethoxybenzene (Fig. 5c) and asparaptine (Fig. 5d). Those variety-specific volatiles were also checked in the second harvest season, and the findings could be confirmed (Fig. 5e, f)

### The potential impact of cultivation method on asparagus metabolome

Modified cultivation methods (e.g. heated field and greenhouse) can be applied to mimic the warm temperature conditions of late spring and thus stimulate the premature development of new spears and enable harvesting earlier in the year (January–March). In the first season, we included samples harvested from a greenhouse (Fortems) and a heated field (Backlim) in early March (Table S1a). The metabolite profiles of these spears appeared to be similar to those of the same varieties harvested in late May from ‘mini-tunnel’ (Fortems) and ‘open fields’ (Backlim) (Supplementary Information; Fig. S1c–e; red arrows). During the second harvest season in 2020, we included additional harvest weeks of the heat-enhanced fields (Fortems greenhouse and Backlim heated field). Again we observed that the volatile profiles of the spears grown under heat-enhanced conditions were similar to those of spears harvested later in the season from ‘mini-tunnel’ (Supplementary Information; Fig. S3; yellow circles) or ‘open field’ plots (Supplementary Information; Fig. S3; blue circles). Although, some variation could be observed when examining individual compounds (Supplementary Information; Fig. S4), the overall profile (Supplementary Information; Fig. S3) and in particular metabolites which have previously been proposed to contribute to asparagus flavour did not significantly vary despite the interval of more than two months between the harvests of spears grown in heat-enhanced conditions and those in the regular fields.

## Discussion

White asparagus is quite a unique crop as the vegetable is harvested while the developing spears are still fully submerged underground. The dynamics of the asparagus metabolome, in association with the growing conditions and the moment of harvest, has to our knowledge never been fully investigated. A valuable start was recently made for green asparagus where changes in levels of specific primary metabolites, total phenolics and minerals throughout the season were monitored (Soteriou et al., 2021). Asparagus chemical composition with regards to flavour and specific health benefits has also been the focus of a small number of studies. Some of these focused on specific aroma compounds (Hoberg et al., 2003, 2008) and bitter, non-volatile compounds (Dawid & Hofmann, 2012, 2014) while others specifically studied the properties of the sensory-relevant sulphur-containing metabolites (Miyoshi et al., 2018; Nakabayashi et al., 2015, 2021; Yanagawa et al., 1972). However, all these reports only partly discussed, if at all, the potential influence of genetic background or harvest history of the asparagus materials on their chemical composition.

In the study presented here, we aimed to explore which chemical shifts occur during the harvest season with respect to the profiles of both the volatile and non-volatile secondary metabolites, given that the majority of these compounds can be relevant for quality traits—and in particular—for the typical asparagus flavour (Pegiou et al., 2020). Our findings highlight the dynamic character of the chemistry of the asparagus crop. This metabolic dynamism during the crop season, reflected by the varying composition of monoterpenes, benzenoids and saponins, might be explained by the rate of spear development, conditions at the precise moment of harvest and the genetic background. However, with respect to flavour quality the overall picture is mainly one of stability regarding the composition of key odorants, suggesting the production of quite a uniform crop, irrespective of genotype or environment. Subtle variations in bitterness is however possible and subtle effects on flavour due to perturbations in individual sensory-relevant compounds cannot be excluded.

### The composition of monoterpenes, volatile benzenoids and saponins during the asparagus season

While the asparagus season lasts almost four months, the actual harvest period of each production field is just 8 weeks. Asparagus has been bred to create both early and late varieties with the former being able to start producing spears in the early months of the season while the latter performs best in the later months when temperature fluctuations are usually less extreme and average temperatures are higher (Pegiou et al., 2020). Here, we have shown that monoterpenes and benzenoids (clustered in C1) in white asparagus spears harvested between late March and early May (early season) decrease along the harvest period, while the same compounds slightly increased towards the end of the season for late varieties harvested between May and mid June (Fig. 3a; Table 1). Interestingly, these monoterpenes and benzenoids also clustered in the V2 of the HCA where a clear differentiation between all the analysed early and late varieties was observed (Fig. 1a). Previous studies have investigated the composition of these secondary plant metabolites with respect to plant growth and development as well as in response to environmental conditions such as light and temperature. Regarding plant monoterpene composition, most studies monitor the emissions of these volatile compounds, thus a direct conclusion for accumulation in the plants cannot be driven. Zawilak (2013) actually studied the content of monoterpenes and sesquiterpenes in essential oil of *Hyssopus officinalis* L. at various developmental stages i.e. vegetative stage in June, beginning of flowering in July and full flowering in August. Monoterpenes were found to significantly decrease in the latest plant growth stage compared to vegetative stage. Whether this was due to increasing temperature, it was not examined, but in our specific case, the average air temperature in mid-May 2019, during the last 3 weeks of the harvest of early varieties was actually significantly lower compared to the previous weeks (11 °C compared to 21 °C) (source: timeanddate.com, weather in Helden, the Netherlands). Similarly in 2020, the average temperature in calendar week 18 was 10 °C and significantly lower than in week 15 when it was 22 °C (source: timeanddate.com) and again a decrease in the abundance of monoterpenes was observed (Fig. 3c). This fall in temperature might therefore explain the observed decrease in monoterpene content. Cheng et al. (2016) used petunia flowers, which is a model to study the regulation of the phenylpropanoid pathway and the biosynthesis of benzenoids in plants and they investigated the sensitivity of volatile benzenoids to varying light and temperature regimes. They showed that both short (e.g., 10 h) and long-term (1 month) exposure of the plants to high temperatures (i.e. 22–28 °C instead of 16 °C) reduced the content of volatile benzenoids as analysed by SPME GC–MS using similar settings as used in this study. Therefore, the lower content of these compounds observed in asparagus at specific times might be explained by the varying temperatures that can occur during the harvest season. Although practically challenging, a controlled lab-type experiment would be needed to confirm this.

Saponins, which are widely distributed in the plant kingdom and are especially prevalent in the *Asparagaceae*, are products of isopentenyl pyrophosphate synthesis via the mevalonate pathway. Their composition in plants can be influenced by genetic background, developmental stage and spatial differential expression (Upadhyay et al., 2018). In *Asparagus* species, steroidal saponins are generally found to be more prevalent in the roots (Srivastava et al., 2018) and the basal parts of the spears (Lee et al., 2010; Dawid & Hofmann, 2014) also reported a decreasing gradient of saponin levels from the base to the tip of asparagus spears and these observations are likely related to their proposed function as underground protectants. The biosynthesis of asparagus saponins has not yet been investigated, but in this study the levels of saponins detected in white spears were found to decrease as the harvest season progressed (Fig. 3b; Table 1). This might suggest that the plant need for saponins decreases later in the season. Alternatively, it may indicate that synthesis of saponins lags behind growth as the rate of the latter increases with rising temperature. Phrompittayarat et al. (2011) studied the content of saponins in *Bacopa monnieri* which is also a perennial plant, and they detected higher saponin content in older (4-month old) plants compared to plants that were 1-month old, while all were grown in summer months. Our findings may also indicate a relationship between the developmental age of the harvested asparagus spear and the saponin content.

The observed patterns for the saponin content in the asparagus spear and all other metabolites clustered in C2 (which also clustered in N2 of Fig. 1b—turquoise box) were notably differentiating for Gijnlim compared to the other varieties examined (Fig. 3b; Table 1). However, this Gijnlim-specific pattern was not observed in the second season (Figs. 3c, d and S4) even though the other prominent temporal (Figs. 3, S2, S3, S4) and varietal (Fig. 5) patterns were again visible. Variation between seasons of some asparagus varieties has been discussed previously with respect to spear physiology and yield (Creydt et al., 2018; Heißner et al., 2006), and our results therefore suggest that the synthesis/accumulation of metabolites may be differentially affected. Variation between seasons might occur for example due to environmental factors or varying growing conditions and this may impact certain synthetic pathways differently. However, climatically, both seasons (2019 and 2020) were essentially representative and involved no extreme weather conditions and contrasts between them.

### Weighted correlation network analysis to unravel complex biological relationships

The temporal changes of the detected compounds during the harvest period have illustrated the complexity and dynamic character of the metabolome of white asparagus spears. No group of metabolites from all tested varieties was found to follow a similar general pattern along the season (Fig. 1). To unravel this heterogeneity and potential compositional shifts, we applied WCNA to cluster the fitted trends that significantly changed over time and used a subset of data from the three varieties which had been harvested at least at 6 time-points. WCNA (or WGCNA) has often been used in ‘omics’ studies, where it has been shown to have great potential for clustering features (e.g. genes, transcripts, peptides, metabolites) which are of biological relevance (Dekker et al., 2022; DiLeo et al., 2011; Ding et al., 2019). This is due to the topological overlap being used for dissimilarity measure in addition to correlation (Langfelder & Horvath, 2008; Pei et al., 2017) and thus indirect relationships between traits, which are common in biological systems, are also taken into account.

WCNA of the significant seasonal metabolite trends highlighted the dynamic character of the asparagus metabolome. The most prominent seasonal changes were found in metabolites assigned to either clusters 1 or 2 (which together comprised ca. 20% of all originally detected compounds) as discussed above in Sect. 4.1. Ninety-one compounds were clustered in the other five clusters of WCNA (C3–C7). The changes for the majority of these compounds were small-close to or less than 2-fold relative to the start of the harvest (Fig. S2). These relatively small temporal changes imply a small impact of time on the composition of these specific asparagus metabolites (Fig. S2), as was also suggested previously (Pegiou et al., 2021). These metabolites are chemically quite diverse and comprised medium-chain carbonyls, furans, phenolics and sulphur-containing compounds (Table 1). Several of these are relevant for specific asparagus biochemistry, such as asparagusic acid (Mitchell & Waring, 2014) and particularly its flavour, including for example dimethyl sulphide, methional and hexanal (Ulrich et al., 2001).

### Potential flavour dynamics during the asparagus season

With regard to white asparagus flavour which is one of the most important quality traits, focus in the past has been placed mainly on cooked materials. This work has helped identify a list of potentially key asparagus odorants (Hoberg et al., 2003; Ulrich et al., 2001). However, there has been no previous study investigating the impact of cultivation dynamics on the volatile profile of the raw vegetable, even though this is determinant to the flavour of the final cooked products. For this reason, we specifically looked at a subset of data for the known sensory-relevant asparagus metabolites. Most changes in individual metabolites were ≤ 2-fold compared to the first harvest (Fig. 4). Moreover, high similarities between the three genotypes studied were found for the key sulphur and nitrogen containing aroma compounds. These findings suggest that there is likely to be no large seasonal or varietal differentiation with regards to asparagus flavour. However with respect to one specific sensory attribute, bitterness, our findings do suggest some variation during the season and potentially also between varieties may occur. The detected bitter compounds shatavarin IX (Onlom et al., 2017), furostane-3,22,26-triol (Dawid & Hofmann, 2012) as well as other saponins did significantly change throughout the season and varied across varieties (Fig. 4 and Supplementary Information; Table S4).

### The impact of heat-enhanced cultivation

Asparagus growers have developed innovative cultivation methods to expand the crop season and allow asparagus spears to develop and enter the market earlier. For white asparagus, which is harvested while still below ground, opaque plastic foil is used to cover the ridges to avoid any exposure of the young spears to sunlight. Furthermore, the use of an additional plastic cover is also often used especially during the first half of the standard production season to create what is known as a mini-tunnel (Pegiou et al., 2020). This mini-tunnel creates a greenhouse-type microclimate which keeps the temperature around the asparagus bed more stable and slightly higher than the ambient weather conditions. This protects the plants during cold days and generally stimulates growth. Greenhouses can also be used where temperatures are artificially controlled-again to allow the harvest of standard early varieties, even earlier, already in the winter. Following similar principles, circulating warm water using a network of tubing under the root crowns is also exploited in heated fields to cultivate selected varieties and again enable harvesting artificially early in the season. However, following standard cultivation practices, May–June is considered to be the “high asparagus season” characterized by higher yields of better quality spears when compared to those obtained at the first harvest in March (Heißner et al., 2006).

To investigate the comparability of asparagus spears obtained via standard and heat-enhanced cultivation methods a range of materials was assessed. Heat-enhanced cultivation methods (Fortems greenhouse, Backlim heated field) were seen to yield asparagus spears with similar metabolite compositions to spears harvested from regular fields during the “high asparagus season” (Fig. S1c–e). Similar conclusions could be made for the second season’s materials (Fig. S3). Flavour-relevant compounds and typical asparagus metabolites were observed to be present at similar levels from both cultivation methods. It is therefore proposed that enhancing the cultivation temperature is able not only to speed up the development process effectively, to deliver spears earlier, but also this delivers spears of equivalent quality to those obtained later in the season using standard field cultivation conditions.

## Supplementary Information

Below is the link to the electronic supplementary material.

Supplementary file1 (DOCX 1393 kb)—Supplementary Tables S1: Harvest schemes for the collected asparagus spears included in this study; S2: detected volatiles with annotation information; S3 detected non-volatiles with annotation information; S4: List of volatile and non-volatile compounds that were significantly different between varieties and/or time-points. Supplementary Figures S1: PCA score plots of the GC-MS and LC-MS data of 2019; S2: Heatmaps of WCNA clusters C3–C7 and time-trends of selected representative metabolites; S3: PCA score plots of the GC-MS data of 2020; S4: Time-trends of selected compounds detected in spears harvested in 2020 season.

## Data Availability

The datasets generated during and/or analysed during the current study are available from the corresponding author on reasonable request.

## Software availability

This article does not contain any new software developed.

## Declarations

### Research involving human and/or animal participants

This article does not contain any studies with human and/or animal participants performed by any of the authors.
