# Supplementary material for: Unravelling the seasonal dynamics of the metabolome of white asparagus spears using untargeted metabolomics
Source: Metabolomics. 2023 Mar 27;19(4):23. doi: 10.1007/s11306-023-01993-0 (PMC10042981; doi:10.1007/s11306-023-01993-0)
Supplement: Supplementary file 1 — Supplementary file1 (DOCX 1393 kb)—Supplementary Tables S1: Harvest schemes for the collected asparagus spears included in this study; S2: detected volatiles with annotation information; S3 detected non-volatiles with annotation information; S4: List of volatile and non-volatile compounds that were significantly different between varieties and/or time-points. Supplementary Figures S1: PCA score plots of the GC-MS and LC-MS data of 2019; S2: Heatmaps of WCNA clusters C3–C7 and time-trends of selected representative metabolites; S3: PCA score plots of the GC-MS data of 2020; S4: Time-trends of selected compounds detected in spears harvested in 2020 season. [file 11306_2023_1993_MOESM1_ESM.docx]

**Supplementary Information**

Unravelling the seasonal dynamics of the metabolome of white asparagus spears using untargeted metabolomics

Journal: Metabolomics

Eirini Pegiou ^1^, Jasper Engel ^2^, Roland Mumm ^3^ and Robert D. Hall ^1,3,^*.

^1^ Laboratory of Plant Physiology, Wageningen University and Research, 6700AA Wageningen, The Netherlands; eirini.pegiou@wur.nl, ORCID: 0000-0001-6543-8562

^2^ Biometris, Wageningen Plant Research, Wageningen University and Research, 6700AA Wageningen, the Netherlands, jasper.engel@wur.nl

^3^ Bioscience, Wageningen Plant Research, Wageningen University and Research, 6700AA Wageningen, The Netherlands; roland.mumm@wur.nl; ORCID: 0000-0002-0603-1508; robert.hall@wur.nl ORCID: 0000-0002-5786-768X

* Correspondence: robert.hall@wur.nl (Robert D. Hall)

**Supplementary Figures (Fig. S 1 – 4)**


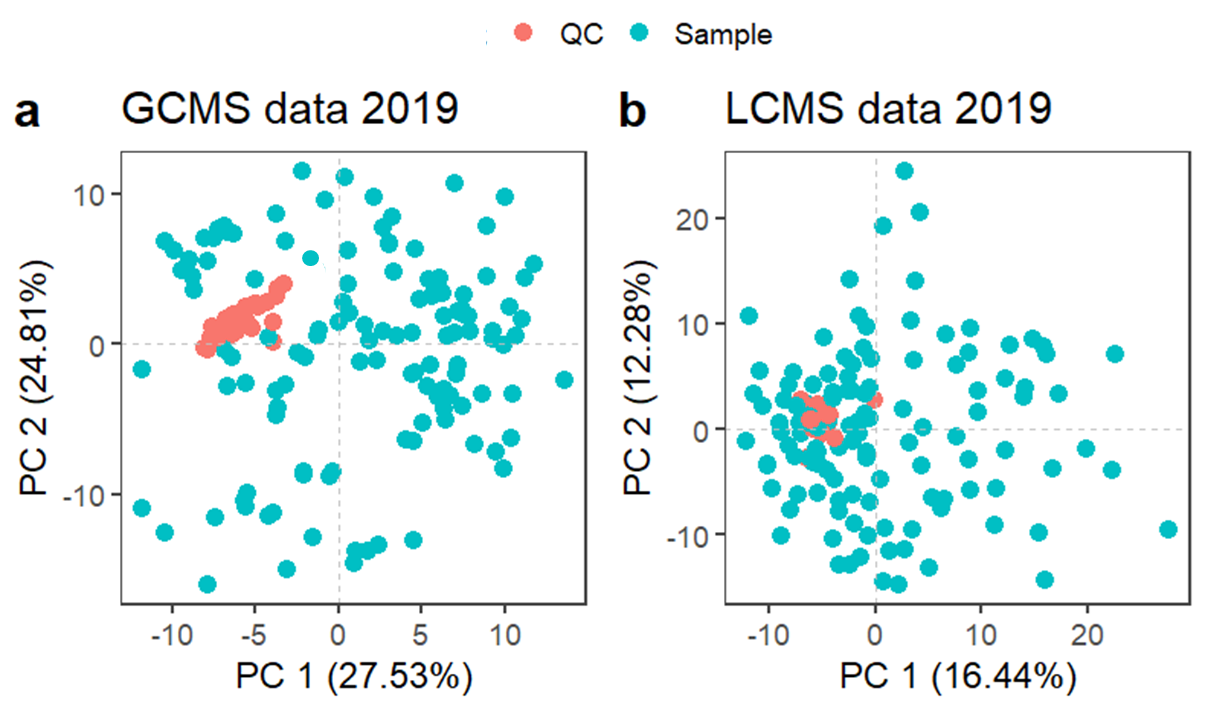


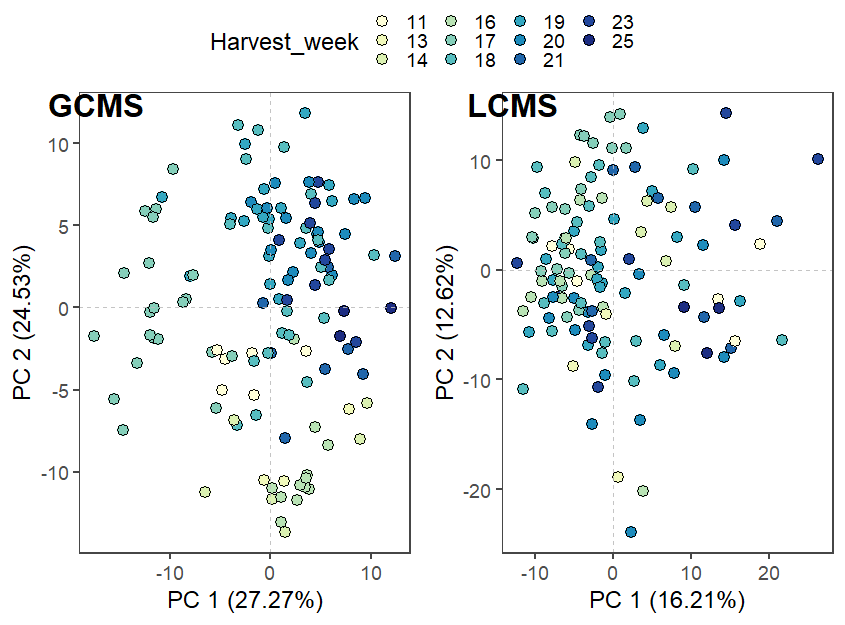


**d**

**c**


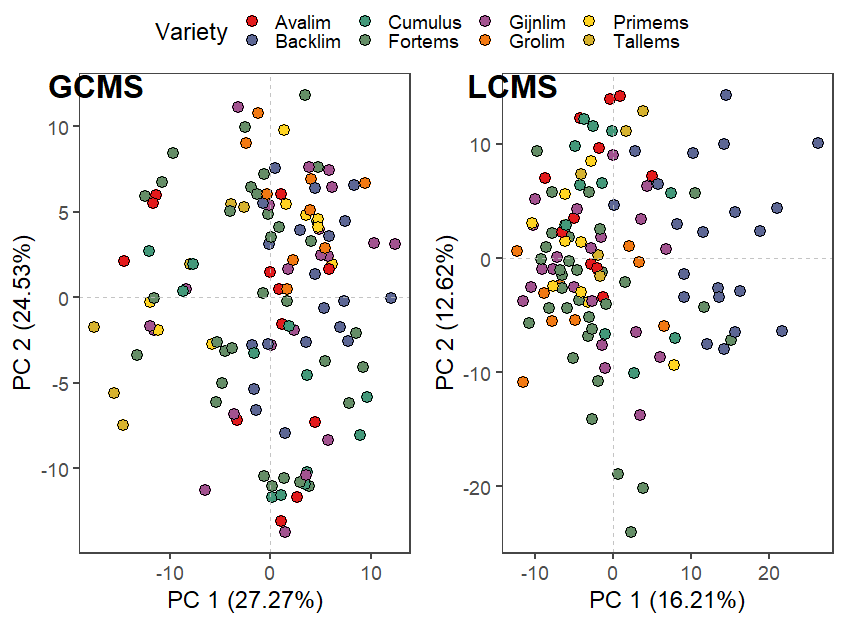


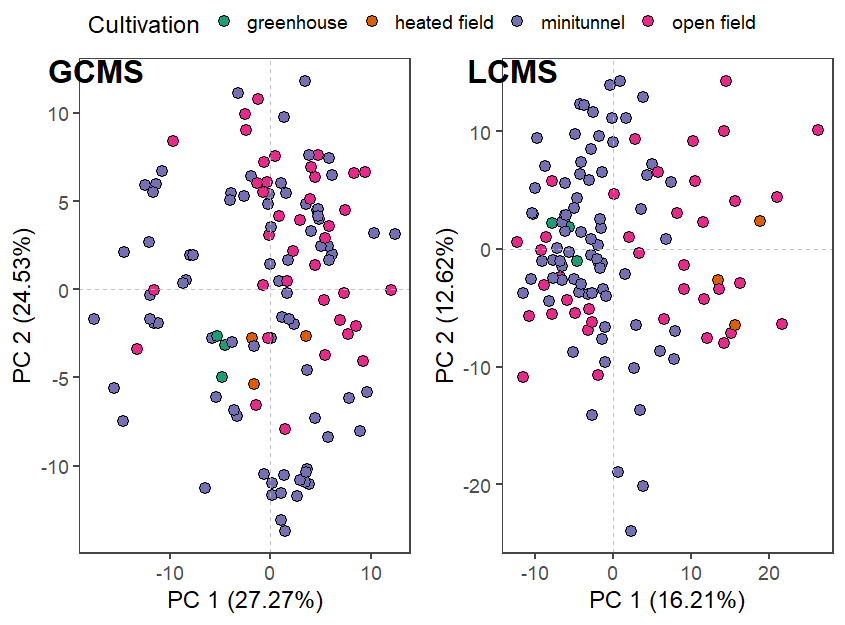


**e**

**Fig. S 1** Principal components analysis of the metabolite profiles of white asparagus spears harvested in 2019. The explained variance per principal component (PC) is shown in parentheses on the axes. Scores of PC1 against PC2 of log2-transformed and pareto-scaled GCMS (**a**) and LCMS data (**b**) including the Quality Control (QC) (red). In the remaining PCA plots after excluding the QCs, data points are colored according to the calendar week throughout the harvest season (**c**), according to variety name (**d**) and according to the cultivation method applied on the fields (**e**). The yellow dotted-line arrow in **c** indicates a possible time-trend in the volatile profiles. Red dotted-line circles in **d** and **e** indicate a possible effect of the variety and cultivation method. Red arrows in **c**, **d**, and **e** highlight the data points of spears grown in heat-enhanced conditions (greenhouse: green, heated field: red) harvested in February/March and of spears grown in mini-tunnels (purple) and open fields (magenta) harvested in May.

**a**


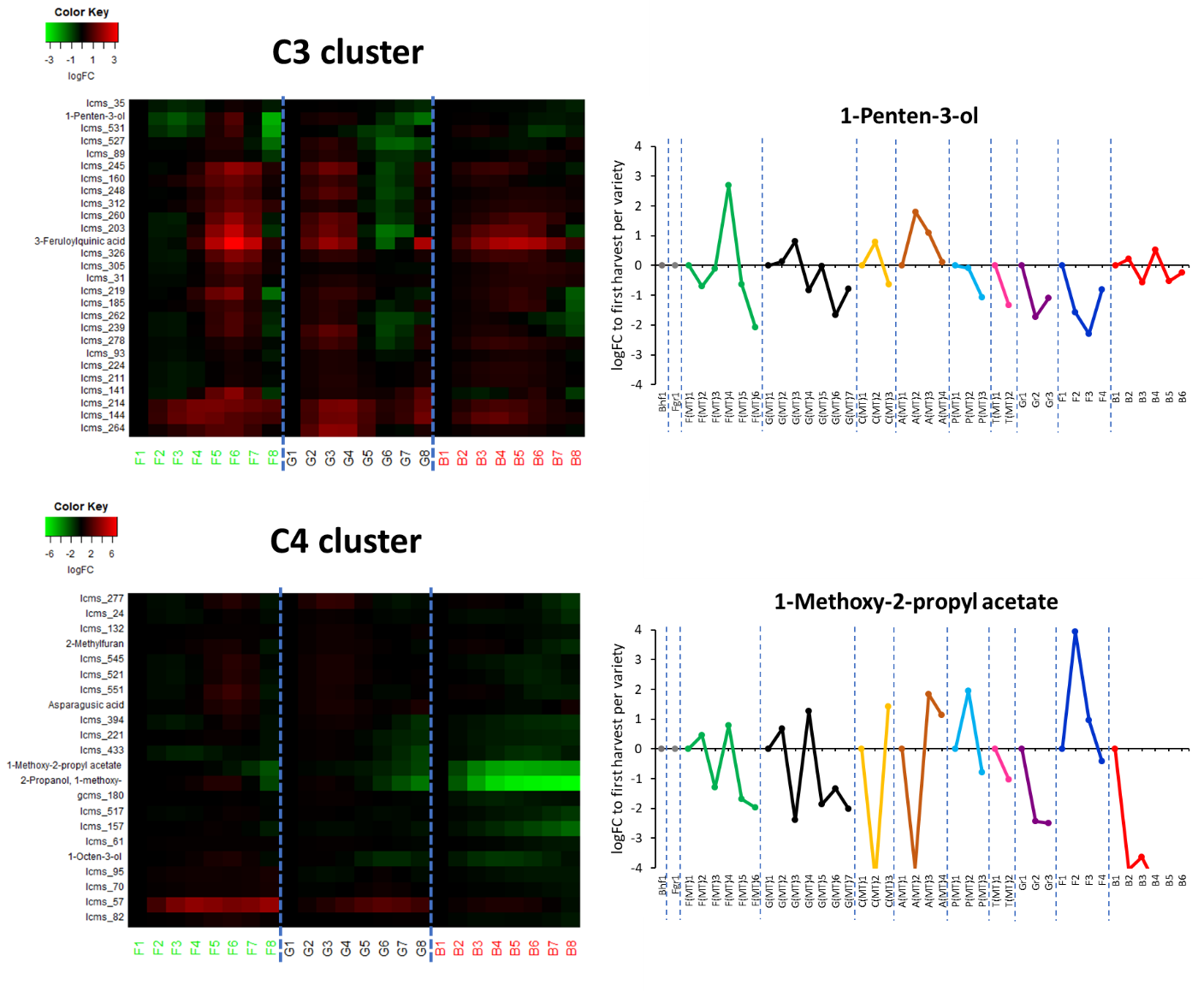

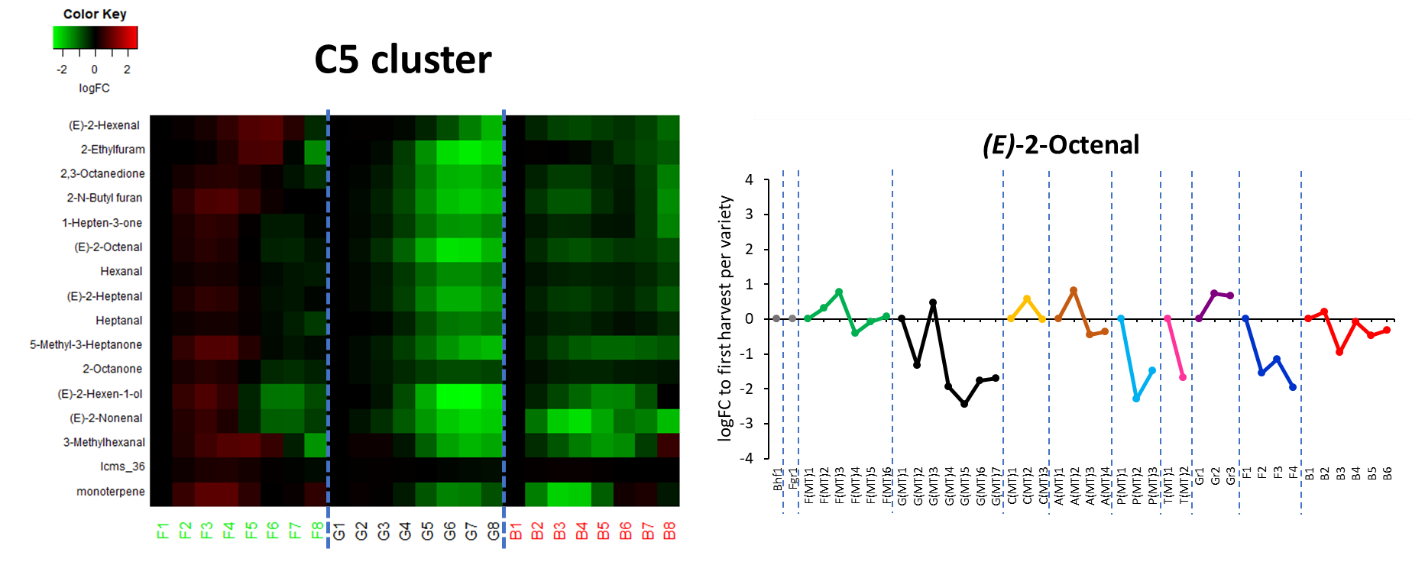


**b**

**c**

**Fig. S 2 (continued in next page)**


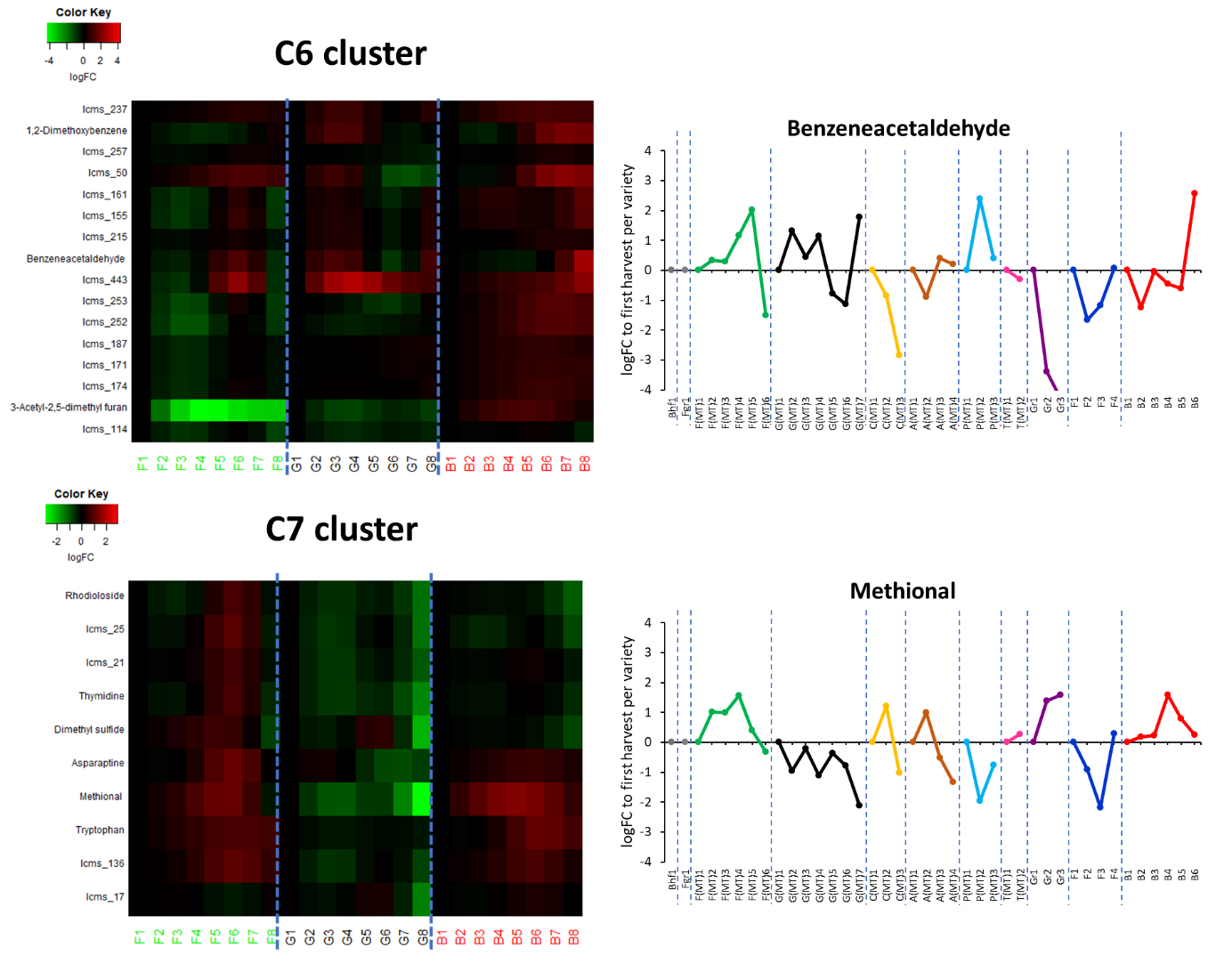


**e**

**d**

**Fig. S 2** Weighted correlation network analysis of the modelled time-trends of metabolites detected in white asparagus spears harvested in 2019. Metabolites were clustered based on their fitted time-trends using topological overlap as a distance measure. Seven clusters were formed (C1 – C7). C1 and C2 are presented in Fig. 3. The time-trends of the clustered metabolites (rows) are visualised in a heatmap (C3: **a**, C4: **b**, C5: **c**, C6: **d**, C7: **e**). Each column represents one harvest week of one variety and varieties are ordered in chronological order of their overall seasonal harvest period. Vertical blue dotted lines separate the harvest periods of the varieties (F: Fortems, G: Gijnlim, B: Backlim). A representative metabolite per cluster was examined with respect to the non-modelled time-trend for all eight varieties which are labelled same as in Fig. 1 (**line graphs**). All clusters are summarized in Table 1.

**b**

**a**


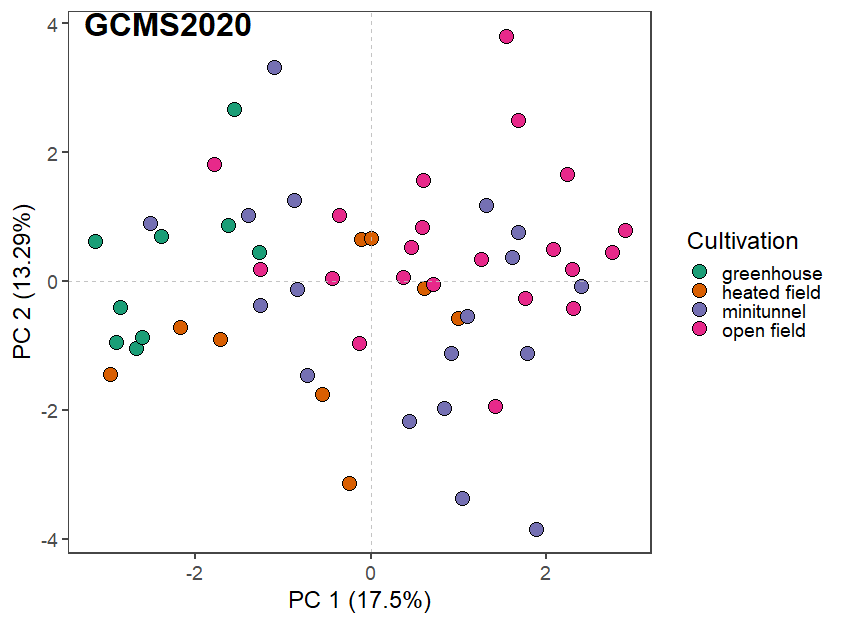

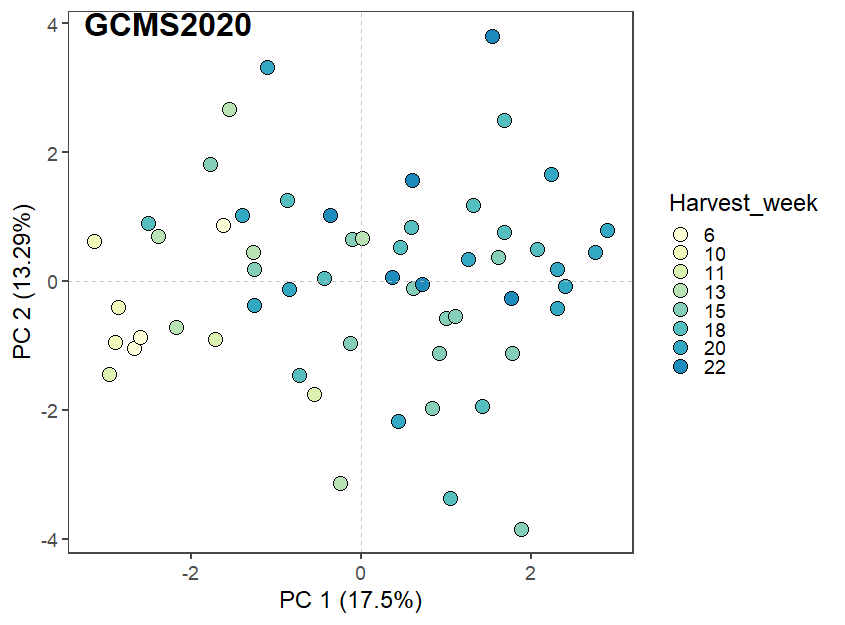


**Fig. S 3** Scores of principal component 1 (PC1) against principal component 2 (PC2) of log2-transformed and pareto-scaled GCMS data of the 2020 harvest season. The explained variance per PC is shown in parentheses on the axes. **a** Data points are colored according to the calendar week throughout the season. **b** Data points are colored according to variety name. **c** Data points are colored according to the cultivation method applied on the fields. Yellow circles indicate possible similarities of the metabolite composition of Fortems harvested early in the season in greenhouse and late in the season from mini-tunnel. Blue circles indicate possible similarities of the metabolite composition of Backlim spears harvested early in the season from heated field and late in the season from regular open field.


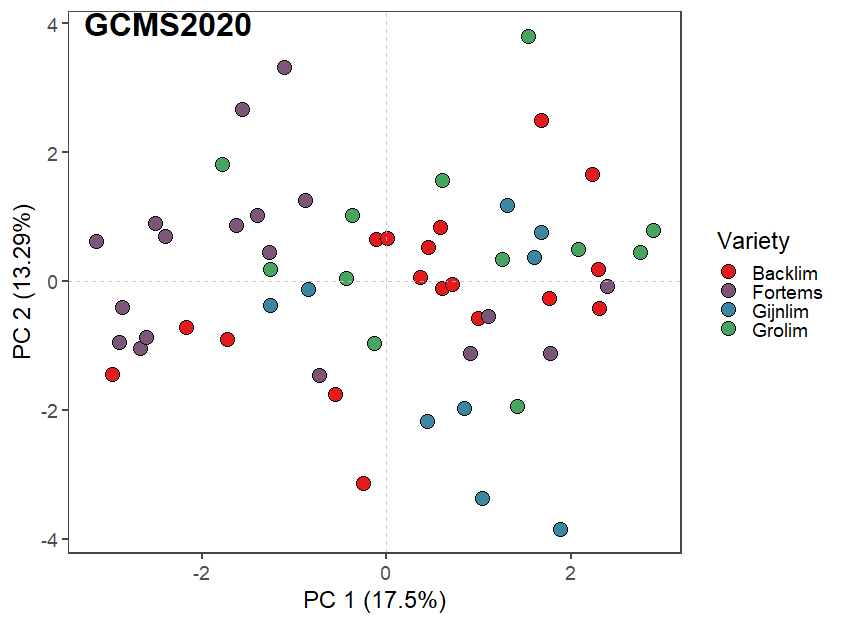


**c**


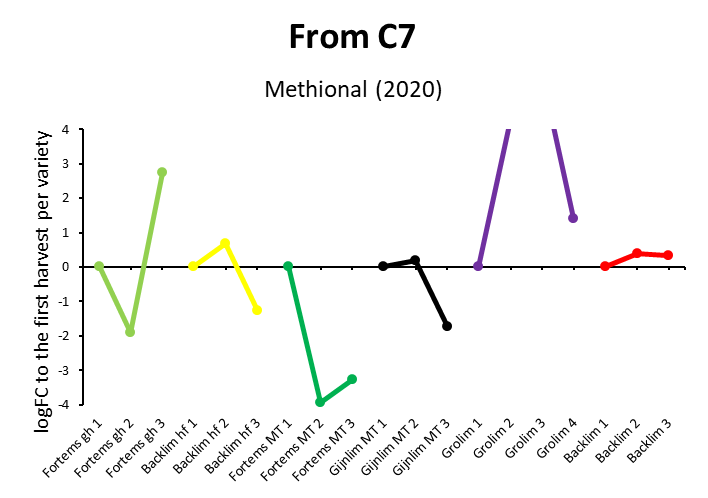

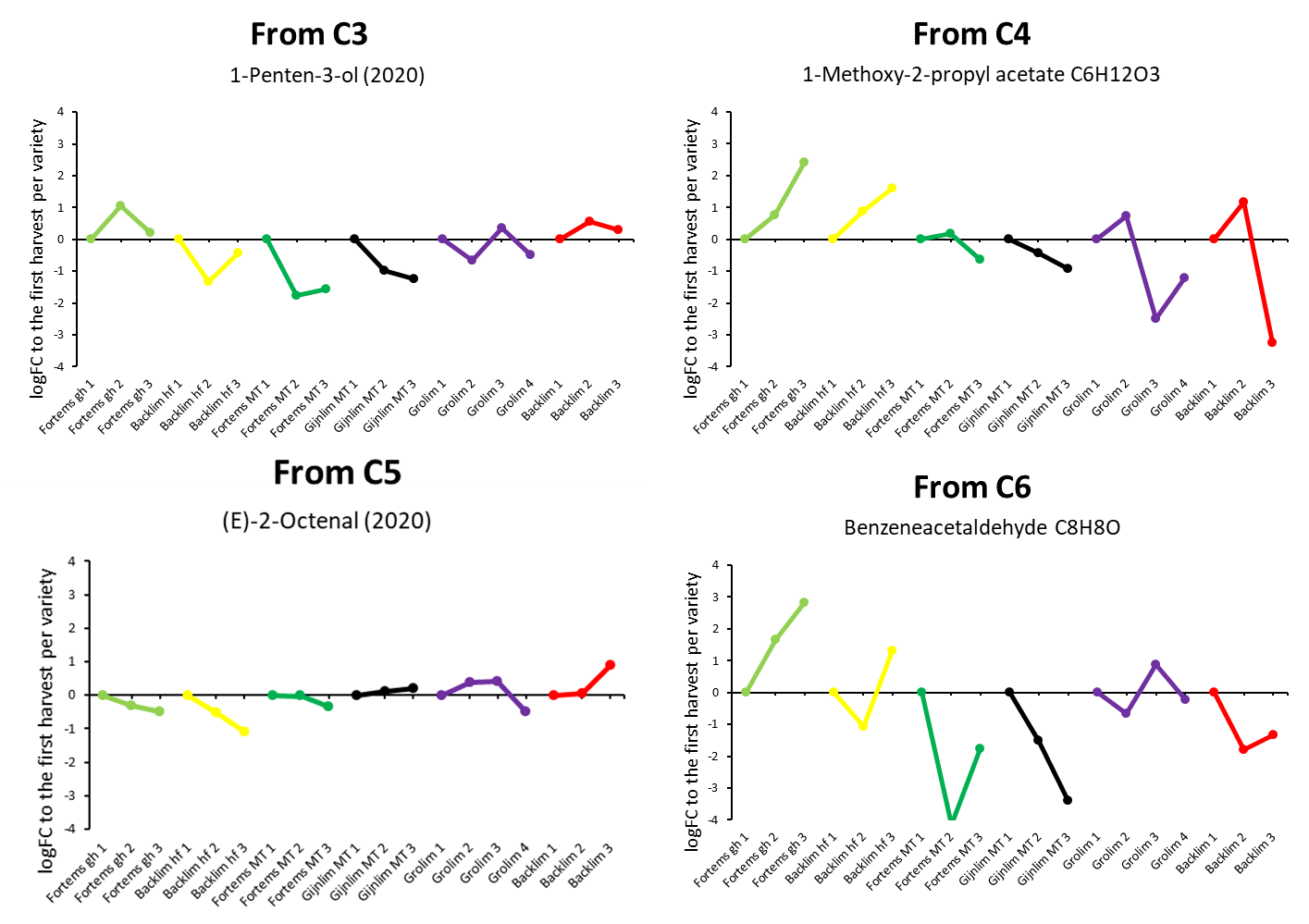


**Fig. S 4** Time-trends of selected metabolites detected in white asparagus spears harvested in 2020. The selected metabolites are representatives of the WCNA clusters presented in Fig. S2. Labels on the x axes indicate the variety name and cultivation method applied in the fields (gh: greenhouse, hf: heated field, MT: mini-tunnel) and numbers indicate the sampled harvest time-point

**Supplementary Tables S1 – S4)**

**Table S1** Harvest schemes for the asparagus spears included in this study. The names of the varieties and the field codes are provided as well as the cultivation method applied (greenhouse, heated field, MT: mini-tunnel, OF: open field); x indicates the calendar week in which spears were harvested. **a** 2019 season. **b** 2020 season.

**a**

| TEBOZA SAMPLES 2019 | | March | | April | | | April/May | May | | | June | |
| --- | --- | --- | --- | --- | --- | --- | --- | --- | --- | --- | --- | --- |
| Varieties | Field/weeks | 11 | 13 | 14 | 16 | 17 | 18 | 19 | 20 | 21 | 23 | 25 |
| Fortems (greenhouse) | Kas 31c | x |  |  |  |  |  |  |  |  |  |  |
| Backlim (heated field) | Smeets | x |  |  |  |  |  |  |  |  |  |  |
| Fortems (MT) | De Meeren 5 |  | x |  | x | x | x | x | x |  |  |  |
| Gijnlim (MT) | Van Zullen 1-3 / Theelen vroeg |  |  | x | x | x | x | x | x | x |  |  |
| Backlim (OF) | Verstegen Schorf |  |  |  |  |  | x | x | x | x | x | x |
| Avalim (MT) | Drissen Vitalim / Elsendorp 2 |  |  |  | x | x | x | x |  |  |  |  |
| Cumulus (MT) | Drissen Vitalim / Elsendorp 2 |  |  | x | x | x | x |  |  |  |  |  |
| Primems (MT) | De Meeren 3 |  |  |  |  | x | x |  | x |  |  |  |
| Tallems (MT) | De Meeren 4 |  |  |  |  | x |  | x |  |  |  |  |
| Grolim (OF) | Veld Will / Theelen laat |  |  |  |  |  | x |  | x |  | x |  |
| Fortems (OF) | Leisink 1 / Lormans |  |  |  |  | x |  | x |  | x | x |  |

**b**

| TEBOZA SAMPLES 2020 | | February | March | | | April | | May | |
| --- | --- | --- | --- | --- | --- | --- | --- | --- | --- |
| Varieties | Field/weeks | 6 | 10 | 11 | 13 | 15 | 18 | 20 | 22 |
| Fortems (greenhouse) | Kas 31c | x | x |  | x |  |  |  |  |
| Fortems (MT) | De Meeren 5 |  |  |  |  | x | x | x |  |
| Grolim | Huis 01 |  |  |  |  | x | x | x | x |
| Backlim (heated field) | Achter loods links |  |  | x | x | x |  |  |  |
| Backlim | Verstegen schorf |  |  |  |  |  | x | x | x |
| Gijnlim (MT) | Theelen vroeg |  |  |  |  | x | x | x |  |

**Table S2** List of volatile compounds detected in white asparagus spears analysed using SPME GC-MS. Annotation information (Putative ID) is provided including calculated retention index (RIexp), chemical formula, CAS number and the level of identification (LOI) assigned following the Metabolomics Standards Initiative guidelines (Sumner et al., 2007). In the case of ‘unknowns’ the base peak is provided in parentheses.

| Compound ID | RIexp | Putative ID | Formula | CAS | LOI |
| --- | --- | --- | --- | --- | --- |
| gcms_2 | 539 | Methanethiol | CH4S | 74-93-1 | 1 |
| gcms_3 | 544 | Ethanol | C2H6O | 64-17-5 | 3 |
| gcms_4 | 558 | Pentane | C5H12 | 109-66-0 | 1 |
| gcms_5 | 559 | Ethyl ether | C4H10O | 60-29-7 | 3 |
| gcms_6 | 565 | Ethyl formate | C3H6O2 | 109-94-4 | 2 |
| gcms_7 | 567 | Dimethyl sulphide | C2H6S | 75-18-3 | 1 |
| gcms_10 | 606 | 2-Butanone | C4H8O | 78-93-3 | 1 |
| gcms_12 | 618 | Ethyl Acetate | C4H8O2 | 141-78-6 | 2 |
| gcms_13 | 618 | 2-Methyl-3-buten-2-ol | C5H10O | 115-18-4 | 1 |
| gcms_14 | 619 | Furan, 2-methyl- | C5H6O | 534-22-5 | 1 |
| gcms_17 | 655 | Butanal, 3-methyl- | C5H10O | 590-86-3 | 1 |
| gcms_18 | 661 | Benzene | C6H6 | 71-43-2 | 2 |
| gcms_19 | 668 | 2-Propanol, 1-methoxy- | C4H10O2 | 107-98-2 | 2 |
| gcms_21 | 677 | 1-Penten-3-ol | C5H10O | 616-25-1 | 1 |
| gcms_22 | 689 | 3-Pentanone | C5H10O | 96-22-0 | 3 |
| gcms_23 | 692 | Pentanal | C5H10O | 110-62-3 | 1 |
| gcms_24 | 694 | Furan, 2-ethyl- | C6H8O | 3208-16-0 | 2 |
| gcms_26 | 700 | (E)-2-Heptene | C7H14 | 14686-13-6 | 2 |
| gcms_27 | 708 | (Z)-3-Heptene | C7H14 | 06/10/7642 | 2 |
| gcms_28 | 727 | 1-Butanol, 3-methyl- | C5H12O | 123-51-3 | 1 |
| gcms_29 | 733 | 4-Methyl-2-pentanone | C6H12O | 108-10-1 | 2 |
| gcms_30 | 737 | unknown (98 base peak) |  |  | 4 |
| gcms_31 | 743 | disulphide, dimethyl | C2H6S2 | 624-92-0 | 1 |
| gcms_35 | 760 | 1-Pentanol | C5H12O | 71-41-0 | 2 |
| gcms_37 | 766 | Toluene | C7H8 | 108-88-3 | 2 |
| gcms_40 | 798 | Hexanal | C6H12O | 66-25-1 | 1 |
| gcms_46 | 808 | Butyl acetate | C6H12O2 | 123-86-4 | 2 |
| gcms_49 | 854 | 2-Hexenal, (E)- | C6H10O | 6728-26-3 | 1 |
| gcms_52 | 861 | 1-Methoxy-2-propyl acetate | C6H12O3 | 108-65-6 | 2 |
| gcms_56 | 867 | Ethylbenzene | C8H10 | 100-41-4 | 2 |
| gcms_57 | 868 | 3-Methylhexanal | C7H14O | 19269-28-4 | 2 |
| gcms_58 | 875 | 1-Butanol, 3-methyl-, acetate (Isoamyl acetate) | C7H14O2 | 123-92-2 | 2 |
| gcms_61 | 876 | Benzene, 1,3-dimethyl- | C8H10 | 108-38-3 | 2 |
| gcms_64 | 886 | 3-Heptanone | C7H14O | 106-35-4 | 2 |
| gcms_65 | 890 | 2-Heptanone | C7H14O | 110-43-0 | 2 |
| gcms_66 | 894 | 2-N-Butyl furan | C8H12O | 4466-24-4 | 2 |
| gcms_68 | 898 | unknown (97 base peak) |  |  | 4 |
| gcms_70 | 900 | Styrene | C8H8 | 100-42-5 | 2 |
| gcms_71 | 902 | unknown (97 base peak) |  |  | 4 |
| gcms_73 | 904 | Heptanal | C7H14O | 111-71-7 | 2 |
| gcms_74 | 911 | Methional | C4H8OS | 3268-49-3 | 1 |
| gcms_76 | 923 | 2,2,4,4-Tetramethyl-3-pentanone (Di-tert-butyl ketone) | C9H18O | 815-24-7 | 2 |
| gcms_77 | 935 | alpha-Thujene | C10H16 | 2867-05-2. | 2 |
| gcms_79 | 946 | alpha-Pinene | C10H16 | 80-56-8 | 2 |
| gcms_82 | 950 | unknown (83 base peak) |  |  | 4 |
| gcms_83 | 962 | 2-Heptenal, (E)- | C7H12O | 18829-55-5 | 2 |
| gcms_85 | 965 | Camphene | C10H16 | 79-92-5 | 2 |
| gcms_86 | 967 | Hexanoic acid | C6H12O2 | 142-62-1 | 3 |
| gcms_88 | 972 | unknown (60 base peak) |  |  | 4 |
| gcms_89 | 972 | unknown (82 base peak) |  |  | 4 |
| gcms_94 | 975 | Benzaldehyde | C7H6O | 100-52-7 | 2 |
| gcms_95 | 982 | 1-Octen-3-one | C8H14O | 4312-99-6 | 2 |
| gcms_97 | 984 | 1-Octen-3-ol | C8H16O | 3391-86-4 | 1 |
| gcms_98 | 985 | Bicyclo[3.1.0]hexane, 4-methylene-1-(1-methylethyl)- (Sabinene) | C10H16 | 3387-41-5 | 2 |
| gcms_99 | 986 | 2,3-Octanedione | C8H14O2 | 585-25-1 | 2 |
| gcms_100 | 990 | Dimethyl trisulphide | C3H6S3 | 3658-80-8 | 1 |
| gcms_101 | 994 | 6-Methyl-5-hepten-2-one | C8H16O | 110-93-0 | 1 |
| gcms_103 | 995 | Î²-Myrcene | C10H16 | 123-35-3 | 2 |
| gcms_105 | 997 | Furan, 2-pentyl- | C9H14O | 3777-69-3 | 1 |
| gcms_107 | 1009 | Octanal | C8H16O | 124-13-0 | 1 |
| gcms_110 | 1019 | 2,4-Heptadienal, (E,E)- | C7H10O | 05/03/4313 | 2 |
| gcms_111 | 1021 | Benzene, (2-methylpropyl)- / Isobutyl benzene | C10H14 | 538-93-2 | 2 |
| gcms_113 | 1023 | Benzene, (1-methylpropyl)- / S-Butyl benzene | C10H14 | 135-98-8 | 2 |
| gcms_115 | 1030 | alpha.-Terpinene | C10H16 | 99-86-5 | 2 |
| gcms_117 | 1038 | Cymene | C10H14 | 527-84-4 | 2 |
| gcms_118 | 1043 | Limonene | C10H16 | 5989-27-5 | 1 |
| gcms_122 | 1047 | Phellandrene | C10H16 | 555-10-2 | 2 |
| gcms_124 | 1051 | 3-(1-Methyl-2-propenyl)-2,4-pentanedione | C9H14O2 | 29149-83-5 | 2 |
| gcms_126 | 1052 | Phenol, 2-propyl- | C9H12O | 644-35-9 | 3 |
| gcms_127 | 1057 | Benzeneacetaldehyde | C8H8O | 122-78-1 | 2 |
| gcms_129 | 1060 | Benzene, 1,3-diethyl- | C10H14 | 141-93-5 | 1 |
| gcms_130 | 1062 | Propanoic acid, 2-(aminooxy)- | C3H7NO3 | 2786-22-3 | 3 |
| gcms_132 | 1066 | 2-Octenal, (E)- | C8H14O | 2548-87-0 | 2 |
| gcms_134 | 1069 | Benzene, 1,4-diethyl- | C10H14 | 105-05-5 | 1 |
| gcms_136 | 1071 | 1,3-Cyclohexadiene, 1-methyl-4-(1-methylethyl)- | C10H16 | 99-86-5 | 3 |
| gcms_137 | 1073 | Benzene, 1,3-diethyl- | C10H14 | 141-93-5 | 3 |
| gcms_139 | 1096 | 2-Nonanone | C9H18O | 821-55-6 | 2 |
| gcms_140 | 1096 | Pyrazine, 2-methoxy-3-(1-methylethyl)- | C8H12N2O | 25773-40-4 | 1 |
| gcms_141 | 1100 | unknown (93 base peak) |  |  | 4 |
| gcms_142 | 1100 | unknown (71 base peak) |  |  | 4 |
| gcms_143 | 1101 | 3,5-Octadien-2-one, (E,E)- | C8H12O | 30086-02-3 | 3 |
| gcms_144 | 1104 | Benzene, 1-methyl-3-(1-methylethenyl)- / 1-Isopropyl-3-methylbenzene | C10H12 | 1124-20-5 | 3 |
| gcms_146 | 1111 | Nonanal | C9H18O | 124-19-6 | 2 |
| gcms_147 | 1115 | 3-Acetyl-2,5-dimethyl furan | C8H10O2 | 10599-70-9 | 3 |
| gcms_152 | 1149 | Benzene, 1,2-dimethoxy- | C8H10O2 | 91-16-7 | 1 |
| gcms_154 | 1167 | 2-Nonenal, (E)- | C9H16O | 18829-56-6 | 2 |
| gcms_155 | 1174 | Pyrazine, 2-methoxy-3-(1-methylpropyl)- | C9H14N2O | 24168-70-5 | 2 |
| gcms_156 | 1193 | Cyclohexanol, 5-methyl-2-(1-methylethyl)-, acetate, | C12H22O2 | 20777-45-1 | 3 |
| gcms_157 | 1196 | 2-Decanone | C10H20O | 693-54-9 | 2 |
| gcms_159 | 1199 | 3-Cyclohexen-1-ol, 4-methyl-1-(1-methylethyl)-, | C10H18O | 20126-76-5 | 2 |
| gcms_165 | 1224 | unknown (43 base peak) |  |  | 4 |
| gcms_166 | 1224 | 2,4-Nonadienal, (E,E)- | C9H14O | 5910-87-2 | 2 |
| gcms_168 | 1244 | unknown (125 base peak) |  |  | 4 |
| gcms_170 | 1250 | 4-Oxononanal | C9H16O2 |  | 2 |
| gcms_171 | 1256 | Nonanoic acid | C9H18O2 | 112-05-0 | 2 |
| gcms_173 | 1268 | 2-Decenal, (E)- | C10H18O | 3913-81-3 | 2 |
| gcms_174 | 1287 | unknown (82 base peak) |  |  | 4 |
| gcms_179 | 1302 | unknown (81 base peak) |  |  | 4 |
| gcms_180 | 1303 | Cyclohexanol, 2-(1,1-dimethylethyl)-, acetate, cis- | C12H22O2 | 20298-69-5 | 3 |
| gcms_181 | 1304 | unknown (95 base peak) |  |  | 4 |
| gcms_183 | 1326 | 2,4-Decadienal, (E,E)- | C10H16O | 25152-84-5 | 2 |
| gcms_185 | 1349 | Unknown (84 base peak) |  |  | 4 |
| gcms_193 | 1419 | unknown (111 base peak) |  |  | 4 |
| gcms_196 | 1455 | unknown (173 base peak) |  |  | 4 |
| gcms_206 | 1595 | Diethyl Phthalate | C12H14O4 | 84-66-2 | 2 |

**Table S3** List of non-volatile compounds detected in white asparagus spears analysed with LC-MS (negative ionisation mode). Annotation information (Putative ID) is provided including retention time in minutes (RT), chemical formula, the calculated monoisotopic ion mass based on the detected base peak and other in-source fragments, and the level of identification (LOI) assigned following the Metabolomics Standards Initiative guidelines (Sumner et al., 2007).

| Compound ID | RT | Putative ID | Formula | [M-H]- | LOI |
| --- | --- | --- | --- | --- | --- |
| lcms_3 | 1.564 |  |  | 272.95941 | 4 |
| lcms_4 | 1.582 |  |  | 288.93695 | 4 |
| lcms_7 | 1.642 |  |  | 372.89822 | 4 |
| lcms_8 | 1.667 | Thymidine | C11H16N2O7 | 287.08521 | 3 |
| lcms_9 | 1.679 |  |  | 242.93993 | 4 |
| lcms_17 | 1.770 |  |  | 238.02347 | 4 |
| lcms_18 | 1.776 |  |  | 112.98581 | 4 |
| lcms_16 | 1.782 |  |  | 380.84372 | 4 |
| lcms_21 | 1.796 | Arginine | C6H14N4O2 | 173.10455 | 2 |
| lcms_20 | 1.814 |  |  | 344.89056 | 4 |
| lcms_24 | 1.827 |  |  | 194.10350 | 4 |
| lcms_25 | 1.846 |  |  | 353.09467 | 4 |
| lcms_28 | 1.888 |  |  | 301.05554 | 4 |
| lcms_29 | 1.916 | Vanillic acid-O-hexoside | C14H18O9 | 329.08679 | 2 |
| lcms_31 | 1.923 |  |  | 269.07809 | 4 |
| lcms_34 | 1.964 | Citrulline | C6H13N3O3 | 174.08868 | 2 |
| lcms_36 | 2.012 | trans-cinnamic acid |  | 147.04926 | 3 |
| lcms_35 | 2.017 | aspartic acid |  | 132.03049 | 2 |
| lcms_39 | 2.035 |  |  | 302.10147 | 4 |
| lcms_41 | 2.069 |  |  | 473.16339 | 4 |
| lcms_42 | 2.106 |  |  | 362.10938 | 4 |
| lcms_43 | 2.107 |  |  | 179.05649 | 4 |
| lcms_47 | 2.133 | Trehalose |  | 341.11414 | 3 |
| lcms_50 | 2.160 |  |  | 157.03699 | 4 |
| lcms_51 | 2.173 | Raffinose |  | 503.16846 | 3 |
| lcms_52 | 2.177 |  |  | 535,15295 | 4 |
| lcms_57 | 2.225 |  |  | 358,11771 | 4 |
| lcms_55 | 2.233 | Quinic acid |  | 191.06207 | 3 |
| lcms_59 | 2.239 | 5-(2-Cyanoethyl)-2-mercapto-4,6-dimethylnicotinonitrile | C11H11N3S | 216.05707 | 3 |
| lcms_61 | 2.252 | 1,3,7-Trimethyluric acid | C6H10O8 | 209.06661 | 3 |
| lcms_64 | 2.310 | Glutaconic / Mesaconinc / Itaconic / Citraconic acid | C5H6O4 | 129.02521 | 4 |
| lcms_65 | 2.310 |  |  | 96.96973 | 4 |
| lcms_70 | 2.331 | Glutathione(reduced) | C10H17N3)6S | 306.07681 | 2 |
| lcms_74 | 2.340 |  |  | 543.09003 | 4 |
| lcms_75 | 2.438 |  |  | 637.18451 | 4 |
| lcms_76 | 2.454 | Acefylline (xanthine alkaloid) | C9H10N4O4 | 237.06201 | 2 |
| lcms_77 | 2.488 | https://www.mzcloud.org/DataViewer#Creference5262#T8626#c | | 362.99704 | 4 |
| lcms_81 | 2.605 | 2,5-Dimercapto-1,3,4-tiadiazole | C2H2N2S3 | 148.94684 | 4 |
| lcms_82 | 2.637 | D-fructose-6-phosphate | C6H13O9P | 259.02295 | 2 |
| lcms_84 | 2.641 | Eriodictyol | C15H12O6 | 287.05899 | 3 |
| lcms_85 | 2.661 |  |  | 168.99072 | 4 |
| lcms_89 | 2.696 | Methyl 3-[2-(dicyanomethylidene)hydrazino]-4-[(2-furylmethyl)sulfonyl]thiophene-2-carboxylate | C14H10N4O5S2 | 377.01245 | 3 |
| lcms_90 | 2.716 | 2,6-Naphthalenedicarboxylic acid | C12H8O4 | 215.03308 | 3 |
| lcms_92 | 2.768 |  |  | 164.01195 | 4 |
| lcms_93 | 2.778 | citric acid | C6H8O7 | 191.01959 | 2 |
| lcms_95 | 2.865 |  |  | 306.07681 | 4 |
| lcms_97 | 2.969 |  |  | 277.02347 | 4 |
| lcms_98 | 2.985 |  |  | 338.98837 | 4 |
| lcms_101 | 3.045 |  |  | 515.05524 | 4 |
| lcms_102 | 3.077 |  |  | 288.98731 | 4 |
| lcms_111 | 3.087 |  |  | 194.92766 | 4 |
| lcms_105 | 3.113 |  |  | 450.98816 | 4 |
| lcms_106 | 3.151 |  |  | 206.04907 | 4 |
| lcms_110 | 3.181 |  |  | 128.03528 | 4 |
| lcms_114 | 3.305 |  |  | 663.01550 | 4 |
| lcms_113 | 3.321 |  |  | 335.98840 | 4 |
| lcms_116 | 3.353 |  |  | 201.00395 | 4 |
| lcms_117 | 3.383 |  |  | 641.05750 | 4 |
| lcms_118 | 3.469 | Uridine diphosphate glucose | C15H24N2O17P2 | 565.04730 | 2 |
| lcms_119 | 3.473 |  |  | 402.99551 | 4 |
| lcms_120 | 3.520 |  |  | 606.07483 | 4 |
| lcms_122 | 3.673 |  |  | 238.02150 | 4 |
| lcms_125 | 3.818 |  |  | 777.23224 | 4 |
| lcms_128 | 3.967 |  |  | 297.06488 | 4 |
| lcms_129 | 4.053 |  |  | 536.11310 | 4 |
| lcms_132 | 4.247 | Chelidonic acid | C11H4OS | 182.99370 | 2 |
| lcms_139 | 4.468 | trans-Aconitic acid | C6H6O6 | 173.00931 | 2 |
| lcms_136 | 4.574 |  |  | 164.07195 | 4 |
| lcms_138 | 4.637 | 2-Cyano-3-(3,4,5-Trimethoxypenyl)prop-2-enethioamide | C13H14N2)3S | 277.06848 | 3 |
| lcms_141 | 4.721 | N-(4-Chlorophenyl)-4-[(4-methylphenyl)sulfonyl]-3-nitrobenzamide | C20H15N2O5SCl | 429.03442 | 3 |
| lcms_143 | 4.841 | <https://www.mzcloud.org/DataViewer#Creference3143> | | 392.11325 | 4 |
| lcms_144 | 4.882 |  |  | 135.01227 | 4 |
| lcms_147 | 5.397 | Asparaptine | C10H18N4O3S2 | 305.07446 | 2 |
| lcms_148 | 5.511 |  |  | 524.18628 | 4 |
| lcms_149 | 5.511 | pantothenic acid | C9H17NO5 | 218.10353 | 2 |
| lcms_150 | 5.580 |  |  | 329.08792 | 4 |
| lcms_153 | 5.643 | N-(2-Cyanoethyl)-N'-[3,5-bis(trifluoromethyl)phenyl]-N-tetrahydrofuran-2-ylmethylthiourea | C17H17N3OSF6 | 424.08569 | 3 |
| lcms_156 | 5.810 |  |  | 438.58426 | 4 |
| lcms_155 | 5.821 | n.i. also in (Pegiou et al., 2021) |  | 586.13849 | 4 |
| lcms_157 | 5.903 |  |  | 563.16211 | 4 |
| lcms_158 | 6.034 |  |  | 256.05441 | 4 |
| lcms_159 | 6.145 |  |  | 342.08759 | 4 |
| lcms_160 | 6.200 | Licoflavonol | C20H18O6 | 353.09610 | 4 |
| lcms_161 | 6.232 |  |  | 586.13855 | 4 |
| lcms_162 | 6.285 | L-Tryptophan | C11H12N2O2 | 203.08238 | 2 |
| lcms_165 | 6.444 |  |  | 476.09769 | 4 |
| lcms_166 | 6.580 |  |  | 526.08093 | 4 |
| lcms_168 | 6.868 | 4,4'-Sulfonylbis[2-(prop-2-en-1-yl)phenol] | C18H18O4S | 329.08774 | 3 |
| lcms_171 | 7.025 |  |  | 119.01744 | 4 |
| lcms_174 | 7.083 | Myricetin | C15H10O8 | 317.04681 | 4 |
| lcms_176 | 7.146 |  |  | 282.07382 | 4 |
| lcms_179 | 7.324 |  |  | 473.08545 | 4 |
| lcms_180 | 7.394 |  |  | 127.00383 | 4 |
| lcms_183 | 7.557 |  |  | 489.12845 | 4 |
| lcms_185 | 7.660 |  | C14H14N3O2SCl | 322.05991 | 4 |
| lcms_187 | 7.709 | https://www.mzcloud.org/DataViewer#Creference4213 | C11H12N2O2S | 235.07019 | 4 |
| lcms_193 | 8.013 |  |  | 518.08276 | 4 |
| lcms_198 | 8.228 |  |  | 277.03207 | 4 |
| lcms_199 | 8.263 |  |  | 843.19092 | 4 |
| lcms_203 | 8.419 | Baicalin | C21H18O11 | 445.08502 | 3 |
| lcms_205 | 8.462 |  |  | 525.16144 | 4 |
| lcms_207 | 8.526 |  |  | 681.13861 | 4 |
| lcms_210 | 8.638 |  |  | 215.14047 | 4 |
| lcms_211 | 8.683 | 1,2-Dithiolane-4-carboxylic acid ester I | C10H16O7S2 | 311.03174 | 3 |
| lcms_212 | 8.734 | 2-Isopropylmalic acid | C7H12O5 | 175.06122 | 2 |
| lcms_213 | 8.958 |  |  | 178.02632 | 4 |
| lcms_214 | 8.990 |  |  | 533.04681 | 4 |
| lcms_215 | 9.037 | 1,2-Dithiolane-4-carboxylic acid ester II | C10H16O7S2 | 266.03380 | 3 |
| lcms_217 | 9.070 |  |  | 239.05652 | 4 |
| lcms_219 | 9.158 |  |  | 519.08569 | 4 |
| lcms_221 | 9.222 |  |  | 731.27789 | 4 |
| lcms_222 | 9.331 | 3-Feruloylquinic acid | C17H20O9 | 367.10339 | 2 |
| lcms_224 | 9.458 | 1,2-Dithiolane-4-carboxylic acid ester III | C10H16O7S2 | 266.03380 | 3 |
| lcms_226 | 9.546 | Dihydroxycinnamic acid isomer 1 | C9H8O4 | 179.03876 | 3 |
| lcms_227 | 9.618 |  |  | 715.21039 | 4 |
| lcms_228 | 9.811 |  |  | 355.10355 | 4 |
| lcms_230 | 9.946 |  |  | 421.16446 | 4 |
| lcms_234 | 10.078 |  |  | 327.10901 | 4 |
| lcms_237 | 10.231 |  |  | 295.08585 | 4 |
| lcms_236 | 10.232 |  |  | 355.10336 | 4 |
| lcms_239 | 10.433 |  |  | 427.18265 | 4 |
| lcms_240 | 10.516 |  |  | 551.17725 | 4 |
| lcms_241 | 10.733 | n.i also in GW |  | 681.24573 | 4 |
| lcms_243 | 10.790 |  |  | 365.13901 | 4 |
| lcms_244 | 10.825 |  |  | 551.17725 | 4 |
| lcms_245 | 11.146 |  |  | 341.03696 | 4 |
| lcms_246 | 11.190 | Isolicoflavonol | C20H18O6 | 353.11389 | 3 |
| lcms_248 | 11.407 |  |  | 461.16681 | 4 |
| lcms_249 | 11.514 |  |  | 441.19818 | 4 |
| lcms_251 | 11.652 |  |  | 787.26642 | 4 |
| lcms_250 | 11.659 |  |  | 384.15997 | 4 |
| lcms_252 | 11.873 |  |  | 561.13220 | 4 |
| lcms_253 | 11.937 |  |  | 607.13788 | 4 |
| lcms_255 | 12.101 | Kaempferol glucoside | C33H40O21 | 771.19934 | 2 |
| lcms_257 | 12.281 |  |  | 523.21869 | 4 |
| lcms_258 | 12.341 |  |  | 490.15637 | 4 |
| lcms_260 | 12.403 |  |  | 429.18240 | 4 |
| lcms_262 | 12.541 | S-Methyl-L-thiocitrulline | C7H15N3O2S | 204.09580 | 4 |
| lcms_263 | 12.776 |  |  | 935.09381 | 4 |
| lcms_264 | 12.849 |  |  | 206.08208 | 4 |
| lcms_267 | 12.991 | Rutin, Quercetin 3-o-rutinoside | C27H30O16 | 609.14624 | 2 |
| lcms_268 | 13.146 |  |  | 504.17197 | 4 |
| lcms_270 | 13.222 |  |  | 565.19293 | 4 |
| lcms_273 | 13.230 |  |  | 319.01273 | 4 |
| lcms_271 | 13.250 | 1,2-Dithiolane-4-carboxylic acid (asparagusic acid) | C4H6O2S2 | 148.97392 | 2 |
| lcms_272 | 13.304 | n.i. also in (Pegiou et al., 2021) |  | 521.20306 | 4 |
| lcms_274 | 13.455 |  |  | 321.15591 | 4 |
| lcms_277 | 13.800 |  |  | 358.11392 | 4 |
| lcms_278 | 13.832 |  |  | 264.98441 | 4 |
| lcms_279 | 13.885 |  |  | 202.09082 | 4 |
| lcms_281 | 14.026 |  |  | 441.19830 | 4 |
| lcms_282 | 14.247 |  |  | 523.21869 | 4 |
| lcms_290 | 14.713 | Isorhamnetin-3-rutinoside (narcissin) | C28H32O16 | 623.16199 | 2 |
| lcms_291 | 14.748 | n.i. also in (Pegiou et al., 2021) |  | 519.18744 | 4 |
| lcms_294 | 14.956 |  |  | 279.12378 | 4 |
| lcms_296 | 15.123 |  |  | 719.34186 | 4 |
| lcms_299 | 15.241 |  |  | 579.17188 | 4 |
| lcms_305 | 15.577 |  |  | 173.08202 | 4 |
| lcms_304 | 15.771 |  |  | 160.84195 | 4 |
| lcms_310 | 15.980 |  |  | 497.22391 | 4 |
| lcms_312 | 16.117 |  |  | 175.09766 | 4 |
| lcms_316 | 16.231 |  |  | 179.06049 | 4 |
| lcms_315 | 16.313 |  |  | 173.08202 | 4 |
| lcms_317 | 16.551 |  |  | 577.26813 | 4 |
| lcms_319 | 16.565 |  |  | 477.05368 | 4 |
| lcms_322 | 16.632 | saponin |  | 1063.54041 | 4 |
| lcms_321 | 16.652 |  |  | 577.77008 | 4 |
| lcms_323 | 16.795 |  |  | 850.38232 | 4 |
| lcms_326 | 16.954 |  |  | 207.06636 | 4 |
| lcms_330 | 17.399 |  |  | 433.20770 | 4 |
| lcms_331 | 17.401 | saponin |  | 1095.56000 | 4 |
| lcms_333 | 17.500 |  |  | 467.21375 | 4 |
| lcms_335 | 17.706 | Furostane-3,22,26-triol | C50H84O23 | 1051.53198 | 2 |
| lcms_338 | 17.831 |  |  | 963.48218 | 4 |
| lcms_339 | 17.899 |  |  | 1049.52393 | 4 |
| lcms_341 | 18.029 |  |  | 1109.53992 | 4 |
| lcms_342 | 18.036 |  |  | 406.19885 | 4 |
| lcms_343 | 18.085 |  |  | 209.08202 | 4 |
| lcms_350 | 18.616 |  |  | 1139.51416 | 4 |
| lcms_351 | 18.642 |  |  | 1065.51404 | 4 |
| lcms_358 | 18.720 | saponin |  | 1091.52979 | 4 |
| lcms_355 | 18.757 |  |  | 576.26019 | 4 |
| lcms_357 | 18.796 |  |  | 1193.56104 | 4 |
| lcms_361 | 19.146 | Protodioscin | C51H84O22 | 1047.54529 | 3 |
| lcms_376 | 19.195 | saponin fragment |  | 933.50690 | 4 |
| lcms_371 | 19.197 |  |  | 1033.53040 | 4 |
| lcms_379 | 19.256 | Timosaponin BII | C45H76O19 | 919.49152 | 2 |
| lcms_382 | 19.410 |  |  | 237.02583 | 4 |
| lcms_384 | 19.566 | Azelaic acid; PlaSMA ID-221 | C9H16O4 | 187.09766 | 2 |
| lcms_385 | 19.624 | Shatavarin IX | C45H74O18 | 901.48486 | 2 |
| lcms_392 | 19.784 |  |  | 435.22321 | 4 |
| lcms_393 | 19.917 |  |  | 787.44885 | 4 |
| lcms_394 | 19.920 |  |  | 697.40112 | 4 |
| lcms_396 | 20.036 |  |  | 801.42896 | 4 |
| lcms_400 | 20.085 |  |  | 445.01221 | 4 |
| lcms_402 | 20.099 | n.i. also in (Pegiou et al., 2021) |  | 1177.56311 | 4 |
| lcms_403 | 20.102 |  |  | 949.50177 | 4 |
| lcms_407 | 20.186 |  |  | 347.17157 | 4 |
| lcms_408 | 20.213 | saponin |  | 1119.52441 | 4 |
| lcms_409 | 20.264 |  |  | 1077.54810 | 4 |
| lcms_413 | 20.402 |  |  | 1091.52954 | 4 |
| lcms_416 | 20.623 |  |  | 1121.54016 | 4 |
| lcms_420 | 20.931 |  |  | 1107.56091 | 4 |
| lcms_421 | 21.062 | n.i. also in (Pegiou et al., 2021) |  | 1031.54761 | 4 |
| lcms_422 | 21.155 |  |  | 1063.53223 | 4 |
| lcms_424 | 21.269 |  |  | 741.39087 | 4 |
| lcms_426 | 21.470 |  |  | 1063.53296 | 4 |
| lcms_430 | 21.785 |  |  | 463.21872 | 4 |
| lcms_429 | 21.901 |  |  | 931.49182 | 4 |
| lcms_433 | 22.029 |  |  | 699.38214 | 4 |
| lcms_434 | 22.434 | Rhodioloside D | C16H30O8 | 349.18720 | 3 |
| lcms_435 | 22.481 |  |  | 903.49664 | 4 |
| lcms_437 | 22.680 |  |  | 495.24500 | 4 |
| lcms_443 | 23.315 |  |  | 443.00278 | 4 |
| lcms_445 | 23.431 |  |  | 417.21378 | 4 |
| lcms_446 | 23.588 | n.i. also in (Pegiou et al., 2021) |  | 933.50671 | 4 |
| lcms_447 | 23.746 | S-Adenosylhomocysteine | C14H20N6O5S | 383.11340 | 2 |
| lcms_448 | 24.045 |  |  | 242.17590 | 4 |
| lcms_449 | 24.263 | N,N,4-Trimethyl-3-({[(5-metyl-3-phenyl-4-isoxazolyl)amino]carbnyl}amino)benzenesulfonaminde | C20H22N4O4S | 413.12473 | 3 |
| lcms_452 | 24.549 |  |  | 787.44873 | 4 |
| lcms_454 | 24.707 |  |  | 329.23395 | 4 |
| lcms_455 | 24.765 | n.i. also in (Pegiou et al., 2021) |  | 443.13538 | 4 |
| lcms_457 | 24.806 |  |  | 422.23908 | 4 |
| lcms_460 | 25.076 |  |  | 915.49756 | 4 |
| lcms_463 | 25.410 |  |  | 443.13544 | 4 |
| lcms_467 | 26.014 |  |  | 639.37500 | 4 |
| lcms_468 | 26.059 |  |  | 931.49146 | 4 |
| lcms_471 | 26.200 |  |  | 681.40637 | 4 |
| lcms_473 | 26.402 |  |  | 577.32367 | 4 |
| lcms_477 | 26.783 |  |  | 929.47595 | 4 |
| lcms_478 | 27.026 |  |  | 376.27029 | 4 |
| lcms_480 | 27.182 | Avermectin | C48H72O14 | 871.50000 | 3 |
| lcms_481 | 27.610 | Spirost-5-ene-3,21-diol | C_45_H_72_O_17_ | 883.47595 | 3 |
| lcms_484 | 28.227 |  |  | 690.38025 | 4 |
| lcms_487 | 28.493 |  |  | 362.29184 | 4 |
| lcms_488 | 28.608 |  |  | 771.45331 | 4 |
| lcms_507 | 30.946 |  |  | 293.17581 | 4 |
| lcms_513 | 32.714 |  |  | 420.25012 | 4 |
| lcms_515 | 33.249 |  |  | 915.46051 | 4 |
| lcms_517 | 33.468 |  |  | 713.25977 | 4 |
| lcms_518 | 33.514 | Desglucomusennin | C45H72O16 | 867.48212 | 3 |
| lcms_521 | 33.677 |  |  | 675.36639 | 4 |
| lcms_527 | 34.465 |  |  | 474.26260 | 4 |
| lcms_529 | 34.575 |  |  | 721.36652 | 4 |
| lcms_531 | 35.075 |  |  | 474.26267 | 4 |
| lcms_532 | 35.323 |  |  | 562.31519 | 4 |
| lcms_533 | 35.426 | Spirost-5-en-3-ol | C45H72O16 | 867.48224 | 3 |
| lcms_535 | 35.602 |  |  | 899.46692 | 4 |
| lcms_536 | 36.070 |  |  | 621.43744 | 4 |
| lcms_537 | 36.192 |  |  | 723.38220 | 4 |
| lcms_541 | 36.814 |  |  | 997.50061 | 4 |
| lcms_542 | 37.031 | 1-(9Z,12Z-Octadecadienoyl)-2-hydroxy-sn-glycero-3-phosphoethanolamine | C23H44NO7P | 476.27835 | 2 |
| lcms_544 | 37.374 |  |  | 767.42206 | 4 |
| lcms_545 | 37.786 |  |  | 559.31256 | 4 |
| lcms_546 | 37.835 |  |  | 939.46057 | 4 |
| lcms_547 | 37.894 | 2-linoleoyl-sn-glycero-3-phosphoethanolamine, LPE(18:2) | C23H44NO7P | 476.27829 | 2 |
| lcms_550 | 38.258 | 1-linoleoyl-glycero-3-phosphocholine, Lysophosphatidylcholine(18:2) | C27H52NO9P | 564.33075 | 2 |
| lcms_551 | 38.612 |  |  | 559.31256 | 4 |
| lcms_553 | 39.098 | Spirost-5-en-3-ol | C_39_H_62_O_12_ | 721.42200 | 3 |
| lcms_556 | 39.296 | 2-linoleoyl-sn-glycero-3-phosphocholine, Lysophosphatidylcholine [0:0/18:2(omega-6)] | C27H52NO9P | 564.33069 | 2 |
| lcms_557 | 39.850 | 1-Palmitoyl-2-hydroxy-sn-glycero-3-phosphoethanolamine, 16:0 LYSO-PE | C21H44NO7P | 452.27820 | 2 |

**Table S4** List of (**a**) volatile and (**b**) non-volatile compounds that were significantly different between the three varieties (Fortems, Gijnlim, Backlim) at the start of their harvest periods (‘Variety’), the abundances of which was at least at one time-point significantly different compared to the first harvest moment (‘Time’), and the time-trends of which significantly varied between the three varieties (‘Interaction’). The Venn diagrams for these data are presented in Fig. 2.

**a**

| Effect | Number of compounds | Compounds annotation or ID |
| --- | --- | --- |
| Interaction Time Variety | 13 | 1,3-diethylbenzene, limonene, cymene, ethanol, 1,3-dimethylbenzene, S-butyl benzene, myrcene, gcms_137, phellandrene, gcms_141, 1,4-diethylbenzene, 1-Isopropyl-3-methylbenzene, styrene |
| Time Variety | 2 | Methional, 2-heptanone |
| Interaction Variety | 5 | gcms_147(furan), gcms_124(ketone), 1,2-dimethoxybenzene, gcms_180, 3-methylbutanal |
| Interaction Time | 11 | Isobutyl benzene, butyl acetate, ethylbenzene, 3-methylhexanal, thujene, benzaldehyde, gcms_98(monoterpene), 1-methoxy-2-propanol, gcms_136 (monoterpene), gcms_159, (monoterpene), 4-methyl-2-pentanone |
| Variety | 2 | methanethiol, gcms_30 |
| Time | 26 | 2-octenal, 1-penten-3-ol, heptanal, 2-nonenal, 2-butylfuran, gcms_206, 2-methoxy-3-isopropylpyrazine, 1-methoxy-2-propyl acetate, dimethyl sulphide, 2,3-octanedione, dimethyl trisulphide, 2-hexanal, Di-tert-butyl ketone, 6-methyl-5-hepten-2-one, 3-heptanone, isoamyl acetate, 2-ethylfuran, 2-methoxy-3-isobutylpyrazine, 1-octen-3-one, nonanoic acid, 2-heptenal, 2-methylfuran, toluene, hexanal, 2-decanone, benzene |
| Interaction | 4 | benzeneacetaldehyde, gcms_193, 1-octen-3-ol, 3-methyl-1-butanol |

**b**

| Effect | Number of compounds | Compounds annotation or ID |
| --- | --- | --- |
| Interaction Time Variety | 29 | lcms_187 (C11H12N2O2S), lcms_252 lcms_240 lcms_339 (saponin), lcms_421 (saponin), D-fructose-6-phosphate, lcms_394, Timosaponin BII, Desglucomusennin, S-Methyl-L-thio-citrulline, lcms_468, shatavarin IX, lcms_452 lcms_480 lcms_291 lcms_18 lcms_446 lcms_542 (C23H44NO7P), lcms_237 lcms_331 (saponin) lcms_517 lcms_41 lcms_28, tryptophan, lcms_59 (C11H11N3S), lcms_376 (saponin), lcms_171, myricetin, lcms_17 |
| Time Variety | 18 | lcms_45,7 Isolicoflavonol, lcms_227, Thymidine, lcms_25, lcms_402 (saponin), Furostane-3,22,26-triol, lcms_198 lcms_4, lcms_244, arginine, lcms_136 lcms_413 lcms_393 lcms_39 lcms_221 lcms_473 lcms_114 |
| Interaction Variety | 27 | Spirost-5-en-3-ol, asparaptine, lcms_257 (flavonoid), lcms_312, Licoflavonol, raffinose, lcms_161, lcms_550 (C27H52NO9P), quinic acid, lcms_65 lcms_95 lcms_205, lcms_454 (saponin), lcms_535, lcms_211 (asparagusic acid ester), lcms_156 lcms_426 lcms_253 lcms_420 lcms_350 lcms_274 lcms_278 lcms_228 lcms_76 (alkaloid), lcms_70 (glutathione), lcms_224 (asparagusic acid ester), 2,5-Dimercapto-1,3,4-tiadiazole |
| Interaction Time | 24 | lcms_541, lcms_449 (C20H22N4O4S), chelidonic acid, lcms_358 (saponin), Uridine diphosphate glucose, Vanillic acid-O-hexoside, lcms_443, lcms_282 (flavonoid), lcms_57 lcms_31 lcms_527 lcms_460, aspartic acid, lcms_416 lcms_305 lcms_455 lcms_433 lcms_185, Baicalin, lcms_89 (C14H10N4O5S2), lcms_264 lcms_507 lcms_24, lcms_408 (saponin) |
| Variety | 16 | lcms_195, lcms_371 (saponin), lcms_351 lcms_270 lcms_429 lcms_355, Protodioscin, lcms_294 lcms_396 lcms_106 lcms_341 lcms_422 lcms_9 lcms_16 lcms_488 lcms_122 |
| Time | 19 | Spirost-5-ene-3,21-diol, lcms_42 lcms_43 lcms_3, 2-Isopropylmalic acid, lcms_150 lcms_157, Rhodioloside D, lcms_61 lcms_144, trans-cinnamic acid, lcms_477 lcms_322 (saponin), lcms_277 lcms_219 lcms_214 lcms_281 lcms_243 lcms_20 |
| Interaction | 27 | lcms_210 lcms_222 lcms_241 lcms_521 lcms_215 lcms_251 lcms_110 lcms_158 lcms_271 lcms_448 lcms_326 lcms_217 lcms_85 lcms_93 lcms_245 lcms_272 lcms_551 lcms_120 lcms_260 lcms_239 lcms_467 lcms_403 lcms_141 lcms_155 lcms_531 lcms_545 lcms_248 |
